# Amyloid pathology fingerprint differentiates post-traumatic stress disorder and traumatic brain injury

**DOI:** 10.1016/j.nicl.2018.05.016

**Published:** 2018-06-05

**Authors:** Abdalla Z. Mohamed, Paul Cumming, Hussein Srour, Tamara Gunasena, Aya Uchida, Courtney Nicole Haller, Fatima Nasrallah, for the Department of Defense Alzheimer's Disease Neuroimaging Initiative

**Affiliations:** a Queensland Brain Institute, The University of Queensland, Brisbane, QLD 4072, Australia; b School of Psychology and Counselling and IHBI, Queensland University of Technology, Brisbane, QLD 4059, Australia; c QIMR-Berghofer Institute, Brisbane, QLD 4006, Australia; d School of Medicine, The University of Queensland, Brisbane, QLD 4072, Australia

**Keywords:** Traumatic brain injury, Post-traumatic stress disorder, Alzheimer's disease, Amyloid, PET, Department of Defense Alzheimer's Disease Neuroimaging Initiative (DOD-ADNI)

## Abstract

**Introduction:**

Traumatic brain injury (TBI) and post-traumatic stress disorder (PTSD) are risk factors for early onset of Alzheimer's disease (AD) and may accelerate the progression rate of AD pathology. As amyloid-beta (Aβ) plaques are a hallmark of AD pathology, we hypothesized that TBI and PTSD might increase Aβ accumulation in the brain.

**Methods:**

We examined PET and neuropsychological data from Vietnam War veterans compiled by the US Department of Defense Alzheimer's Disease Neuroimaging Initiative, to examine the spatial distribution of Aβ in male veterans' who had experienced a TBI and/or developed PTSD. Subjects were classified into controls, TBI only, PTSD only, and TBI with PTSD (TBI_PTSD) groups and data were analyzed using both voxel-based and ROI-based approaches.

**Results:**

Compared to controls, all three clinical groups showed a pattern of mainly increased referenced standard uptake values (SUVR) for the amyloid tracer [^18^F]-AV45 PET, with rank order PTSD > TBI_PTSD > TBI > Control, and same rank order was seen in the deficits of cognitive functions. SUVR increase was observed in widespread cortical regions of the PTSD group; in white matter of the TBI_PTSD group; and cerebellum and precuneus area of the TBI group, in contrast with controls. The [^18^F]-AV45 SUVR correlated negatively with cerebrospinal fluid (CSF) amyloid levels and positively with the CSF tau concentrations.

**Conclusion:**

These results suggest that both TBI and PTSD are substantial risk factors for cognition decline and increased Aβ deposition resembling that in AD. In addition, both PTSD and TBI_PTSD have a different pathways of Aβ accumulation.

## 1 Introduction

Post-traumatic stress disorder (PTSD) is an anxiety disorder occurring in response to traumatic stress such as may occur in combat. A retrospective study of t veterans with PTSD showed a two-fold higher risk of developing dementia compared to veterans without PTSD even after adjusting for other comorbidities (Yaffe et al., 2010), and PTSD increases the risk for dementia and Alzheimer's disease (AD) (Cohen et al., 2013; Finfer and Cohen, 2000; Wang et al., 2010; Yaffe et al., 2010b). Traumatic brain injury (TBI) is one of the leading causes of death and disability in the young population (Finfer and Cohen, 2000). Survivors of TBI often suffer from permanent neurological and cognitive deficits, with increased risk for developing AD. There is considerable comorbidity of PTSD with TBI, especially among war veterans (Alway et al., 2016; Amen et al., 2015), which raises the question of whether the two conditions interact regarding the risk of neurodegenerative changes and dementia.

The most common form of dementia is AD, which is characterized by brain pathology consisting of extracellular Aβ plaques, intraneuronal tangles of phosphorylated tau protein, synapse loss, and neuronal loss (Hardy, 2002; Kerbler et al., 2015).The transition from mild cognitive impairment (MCI) to AD can be monitored in PET studies with amyloid-beta (Aβ) ligands such as [^11^C]-PiB and [^18^F]-AV45 (Florbetapir) (Nemmi et al., 2014; Saint-Aubert et al., 2013). Increased Aβ plaque deposition has been reported in post mortem examination of brain from TBI victims (Johnson et al., 2012), and is now being documented in PET studies of TBI patients. For example, in a [^11^C]-PiB PET study of TBI patients, researchers found increased Aβ deposition in the posterior cingulate cortex, cerebellum and striatum (Scott et al., 2016), regions that extensively overlapped with the pattern of Aβ deposition found in AD brain. Indeed, Aβ plaques have been observed in about one third of TBI subjects (Johnson et al., 2012, Johnson et al., 2010), irrespective of patient's age, suggesting that TBI is a causative factor in Aβ deposition (Uryu et al., 2007). Interestingly, Aβ plaques were seen shortly after an injury event (Ikonomovic et al., 2004) but not in long term survivors of TBI (Chen et al., 2009), suggesting that enhanced amyloid clearance mechanisms may compensate for an acute injury-related accumulation.

There is scant documentation of the relationship between PTSD with amyloid deposition. Studies in rodent models of PTSD due to chronic stress indicated accelerated amyloid plaque formation and Aβ release (Rothman et al., 2012). To investigate this phenomenon, the Department of Defense (DOD) funded a component of the Alzheimer's Disease Neuroimaging Initiative (ADNI) specifically to investigate amyloid burden, cognitive changes, and brain structural/functional changes in Vietnam War veterans. A preliminary analysis of data from the DOD-ADNI consortium showed worse global cognitive function in Vietnam War veterans with PTSD, but did not reveal the expected and hypothesized increase in Aβ burden when considering the entire cerebral cortex as a single volume of interest (Weiner et al., 2017). We hypothesized that the earlier analysis of this data may have missed focal alterations in Aβ deposition in the subgroup of long-term PTSD survivors, and furthermore that spatial mapping of Aβ might distinguish the PTSD group from groups with TBI and cases of mixed TBI and PTSD (TBI_PTSD). To test these linked hypothesis, we undertook a voxel-based re-analysis of the DOD-ADNI data sets, and made a statistical comparison of the PTSD, TBI and TBI_PTSD group findings contrasted with demographically matched control material.

## 2 Materials and method

### 2.1 Study subjects

Data used in the preparation of this article were obtained from the DOD-ADNI as part of ADNI database (adni.loni.usc.edu) which was launched in 2003 as a public-private partnership, led by Principal Investigator Michael W. Weiner, MD. The primary goal of ADNI has been to test whether serial MRI, PET, other biological markers, and clinical and neuropsychological assessment can be combined to measure the mild cognitive impairment progression and early onset of AD. DOD-ADNI aimed at investigating the effect of TBI and PTSD on brain function and structure, and identifies the risk for progression to AD. For up-to-date information, see www.adni-info.org.

Ethical approval to use the de-identified data was also obtained from the Human Research Ethics Committee of the University of Queensland, Australia (IRB number #2017000630). Data were collected as part of a multicenter trial run by the DOD-ADNI initiative. In addition, as part of DOD-ADNI, all subjects signed an informed consent form including all the experimental procedures.

**Fig. 1:**
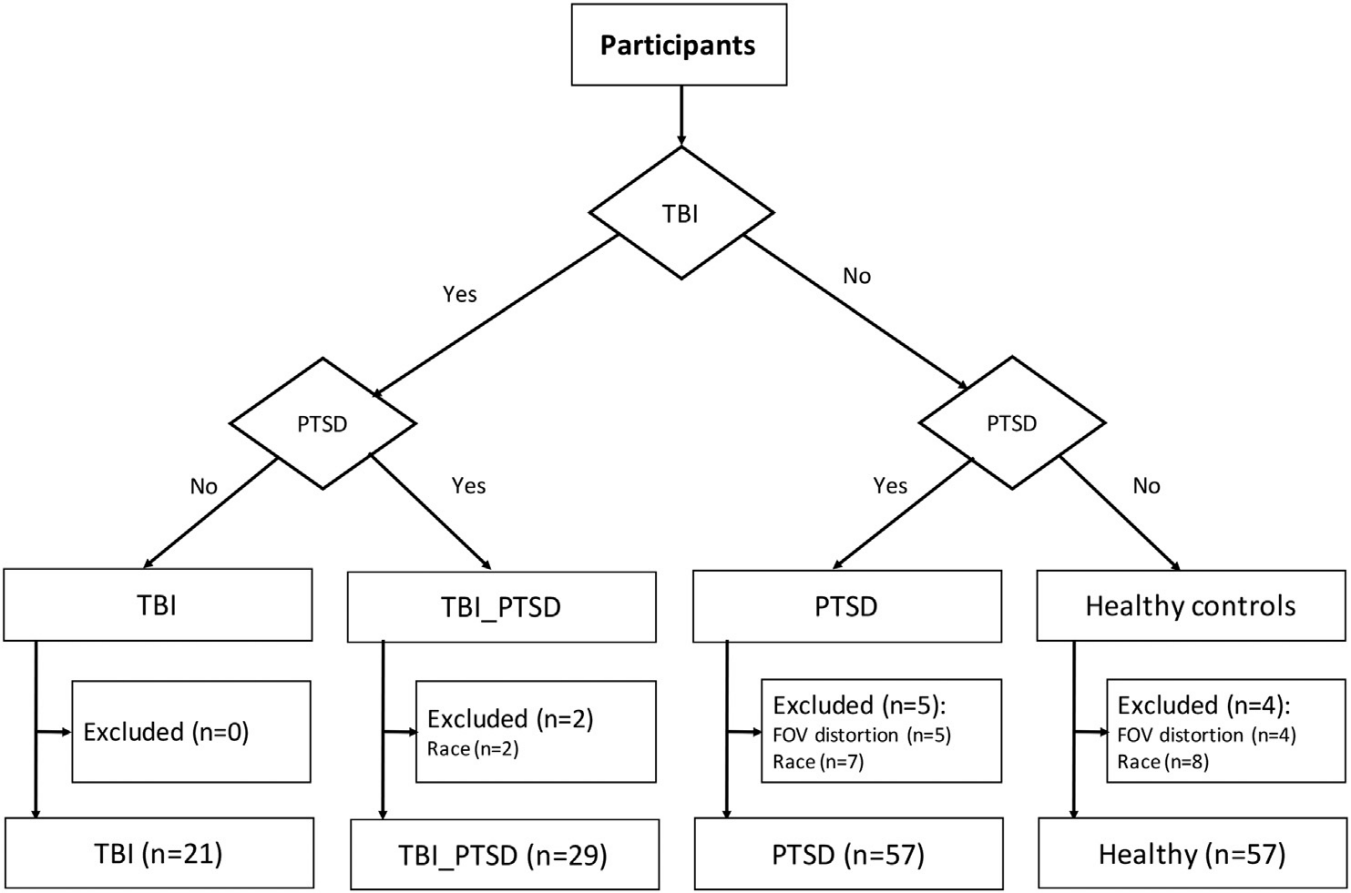
Final groups and clinically classified subjects. Subjects were classified into four different groups including healthy controls, traumatic brain injury (TBI), post-traumatic stress disorder (PTSD), and TBI subjects with PTSD (TBI_PTSD).

A total of 190 male Vietnam War veterans (age = 74.8 ± 6.2 years) were recruited by the DOD-ADNI study. After a rigorous quality control of the PET imaging data for motion, attenuation and other image artifacts, there remained 166 subjects (age 73.7 ± 6.2 years). These individuals belonged to one of four clinical groups: healthy controls without psychiatric or neurological illness (n = 57 subjects), PTSD without TBI (PTSD; n = 57 subjects), PTSD with TBI (TBI_PTSD; n = 29 subjects), and moderate/severe TBI without PTSD (TBI; n = 21 subjects), as shown in Fig. 1. We excluded from our study a total of 17 male veterans of non-Caucasian ethnicity, as shown in Fig. 1. Here, our intention was to eliminate confounds due to possible ethnicity-related differences in brain volume (Isamah et al., 2010) and grey matter integrity (Liu et al., 2015), as shown in Fig. 1. All subjects had served during the Vietnam War with or without documented history of moderate-severe non-penetrating TBI related to military service, and with/without evidence of PTSD identified using the Structured Diagnostic Interview for DSM-V & Clinician Administered PTSD Scale (CAPS) with a cut-off score of 30 (refer to Fig. 1. for further details on the study population).

### 2.2 Study design

The study is a multimodality non-randomized natural history non-intervention study. DOD-ADNI recruited Vietnam War Veterans, identified from the Veteran Affairs Compensation and Pension records, to undergo a multitude of assessments including magnetic resonance imaging (MRI), amyloid PET using [^18^F]-AV45 (Forbetapir), cognitive testing, cerebral spinal fluid biomarkers of tau, phosphorylated-tau, Aβ, and blood sampling for analysis of genetic factors associated with a history of TBI or ongoing PTSD.

### 2.3 Neuropsychological assessment

All participants underwent a battery of cognitive and neuropsychological assessments including Montreal Cognitive Assessment (MoCA) (Nasreddine et al., 2005), Everyday Cognition (ECog) (Farias et al., 2008), Mini-Mental State Exam (MMSE) (Folstein et al., 1975), Alzheimer's Disease Assessment Scale-Cognitive (ADAS-Cog) (Skinner et al., 2012), American National Adult Reading Test (AMEreading) (Spreen and Strauss, 1998), Clinical Dementia Rating (CDR) (Morris, 1993), Functional Assessment Questionnaire (Ito et al., 2012), Combat Exposure Scale (CES), and Geriatric Depression Scale (GDtotal).

### 2.4 Cerebrospinal fluid (CSF) sample collection and analysis

CSF samples were collected through lumbar puncture and were analyzed with established ADNI methods (Aisen et al., 2010) to measure levels of Aβ42, total tau, and phosphorylated tau at threonine 181 (p-tau_181_) using the validated Luminex xMAP multiplex immunoassay platform.

### 2.5 MRI and PET imaging

#### 2.5.1 MRI acquisition

All procedures were standardized across all sites for the conduct and acquisition of the data using qualified ADNI systems. T1-weighted anatomical image was acquired with a Spoiled Gradient Recalled Echo sequence (TE/TI/FA/BW1 = Min/400/11/31.25, sagittal plane, matrix size 256 × 256 × 200 and resolution 1 × 1 × 1.2 mm).

#### 2.5.2 PET acquisition

Amyloid PET images were acquired using [^18^F]-AV45, a radiopharmaceutical that binds to amyloid plaques. A dose of 370 MBq (10 mCi ± 10%) bolus injection of [^18^F]-AV45 was administered to each subject followed by resting-uptake phase of 50 min, whereupon subjects were positioned within the tomograph for acquisition of four emission frames of 5 min each. PET images were 3D reconstructed using Iterative (fully 3D Iteration; four iterations; 20 subsets) with a grid of 128 × 128, FOV: 256 × 256 mm, slice thickness: 3.27 mm.

### 2.6 Image preprocessing and analysis

Data pre-processing and analysis were performed using FMRIB's Software Library (Jenkinson et al., 2012) (FSL 5.0.9, Analysis Group, FMRIB, Oxford, UK, 2012) and Freesurfer (Fischl, 2012) (Martinos Center for Biomedical Imaging, Laboratory for Computational Neuroimaging, Boston, United States).

#### 2.6.1 MRI image processing

MR images were reoriented to match with the Montreal Neurological Institute (MNI) structural template (Grabner et al., 2006) and MRI image were segmented using the *RECON-ALL* function from Freesurfer. The cerebellum of each subject was segmented for the preprocessing of the PET data. Structural data were then resampled to 1.5 mm isotropic resolution and co-registered to the MNI-152 template using nonlinear co-registration tool (FNIRT) (Andersson et al., 2007).

#### 2.6.2 PET pre-processing

The four emission frames were co-registered to each other to correct for head motion, and standard uptake value (SUV) was calculated voxel wise. The SUV images were reoriented to the FSL orientation and resampled to 1.5 × 1.5 × 1.5 mm^3^ to match with the MNI template's resolution. The SUV maps were coregistered to each subject's T1-weighted image using a linear co-registration tool (Flirt) (Jenkinson et al., 2002) to match the subject's SUV map with the individual structural MRI image. To compute the referenced SUV (SUVR) maps, we followed the conventional practice in in scaling the entire brain signal intensity to the mean activity in the individual cerebellum (WH + GM), which was segmented on the MRI. This is a standard approach for transforming SUV maps to normalized SUVR maps for this PET tracer (Scott et al., 2016; Weiner et al., 2017). In particular, we co-registered the SUV maps of each individual to the corresponding segmented T1 structural MRI data, and then extracted the cerebellum SUV value, which was used to calculate parametric SUVR maps of each individual.

The SUVR maps were co-registered to the MNI template using both the transformation matrix and warp files calculated for the T1-MRI registration to the MNI template. The registered SUVR maps were smoothed using a Gaussian filter with a common isotropic resolution of 6 mm and amyloid positivity was defined according to the threshold of SUVR >1.1 (Landau et al., 2012).

### 2.7 Statistical analysis

Neuropsychological data were analyzed using RStudio 3.3.3. Since non-normality was present in the data, we performed nonparametric tests using Kruskal-Wallis tests to examine group differences, followed by performance of pairwise comparisons using Conover-Iman tests and Spearman's rho to examine the correlations between measurements. Statistical significance was set at α = 0.05. Correlations between CSF β-amyloid and tau levels, and SUVR of the parietal, temporal, frontal and cingulate cortices were calculated for each group using Spearman's rho correlation test.

PET SUVR maps were used to generate group's average maps using the one sample *t*-test with FSL-randomize (Winkler et al., 2014). To contrast the amyloid accumulation in clinical groups versus the control group, we used an unpaired 2-sample *t*-test to generate voxel-based difference maps using FSL-randomize nonparametric permutation tests with 1000 permutations. Results were corrected using cluster-based correction through threshold-free cluster enhancement (*p* < 0.05, cluster volume > 40 voxels).

To investigate the group differences in brain regions by VOI analysis, individuals' SUVR values were extracted from a set of templates including frontal, cingulate, parietal, temporal cortices, anterior cingulate cortex, middle temporal gyrus, inferior parietal lobule, medial frontal gyrus and precuneus, all defined in the individual structure MRI data. The group VOI data were analyzed using unpaired *t*-test to identify regions with significant differences between groups, with Bonferroni correction (*p* < 0.05). Correlations between CSF amyloid and tau, and SUVR of the parietal, temporal, frontal and cingulate cortices were calculated for each group using Pearson's r-correlation test (*p* < 0.05).

## 3 Results

### 3.1 Clinical outcome and TBI and/or PTSD groups

**Fig. 2:**
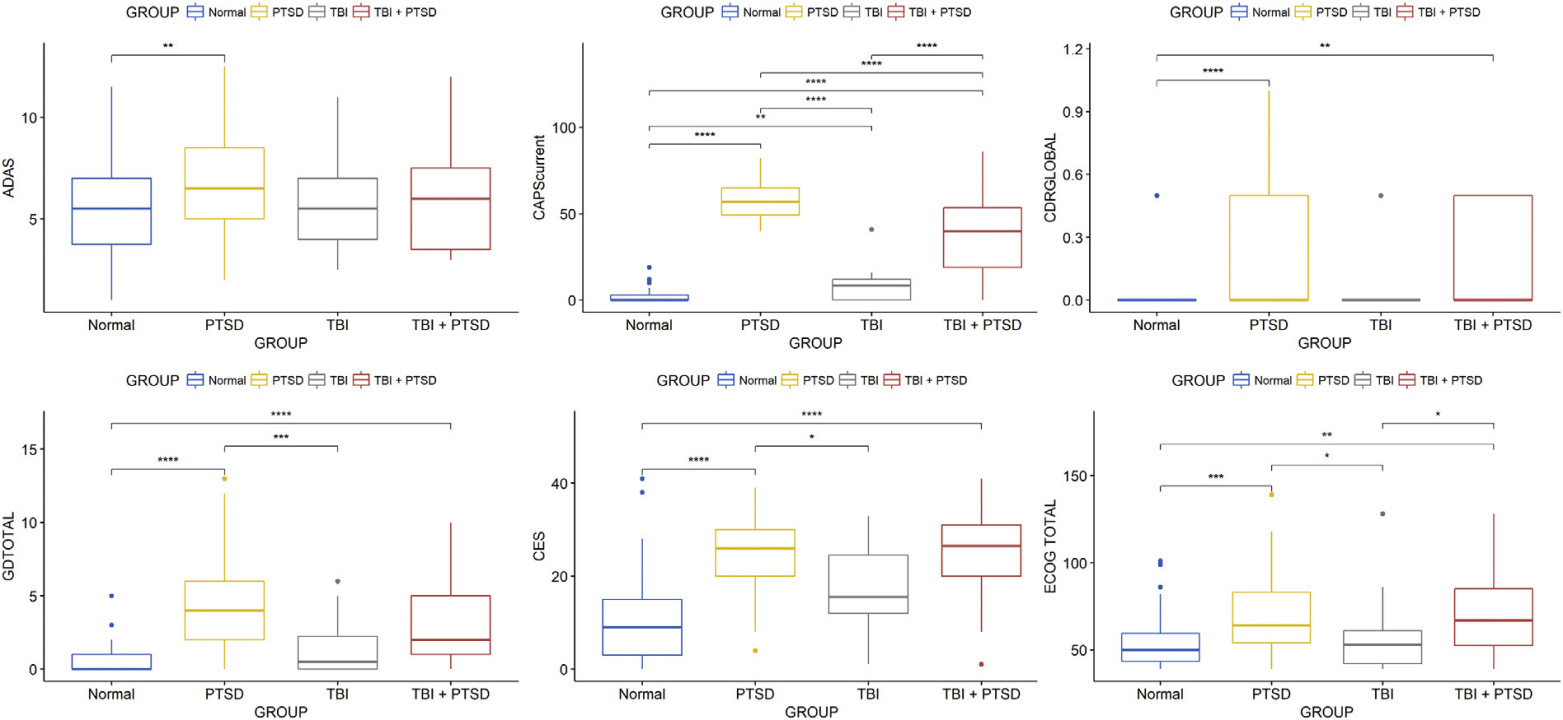
Charts showing the Neurophysiological and clinical outcomes differences. Significant differences were identified with *p* < 0.01. CAPS, Clinician-Administered PTSD Scale; CES, Center for Epidemiologic Studies Depression Scale; CDR, Clinical Dementia Rating; ECOGtotal, total score for Everyday cognition; GDtotal, Geriatric Depression Scale; FSIQ, Full Scale Intelligence Quotient; TBI, traumatic brain injury; PTSD, post-traumatic stress disorder; TBI_PTSD, TBI subjects who developed PTSD.

The mean current-CAPS scores were significantly higher in the PTSD group in contrast to TBI_PTSD (*p* < 0.0001), and both PTSD/TBI_PTSD had higher mean scores than the control (*p* < 0.0001) and TBI group (*p* < 0.00001). The mean CDRGLOBAL score was higher in PTSD compared to controls (*p* < 0.00001) and TBI_PTSD (*p* < 0.001), suggesting that cognitive deficits were more pronounced in both PTSD groups than in the TBI alone or healthy control groups. Mean CES score was significantly higher in the PTSD/TBI_PTSD groups compared to controls (*p* < 0.00001), but only the PTSD group had significantly higher CES scores than did the TBI group (*p* < 0.01). The mean GDtotal score was higher in PTSD/TBI_PTSD groups compared to controls (*p* < 0.0001), and higher in the PTSD compared to the TBI group (*p* < 0.0001). The mean total ECog scores indicated significantly worse cognition in the PTSD/TBI_PTSD groups compared to the control (*p* < 0.0001, *p* < 0.001) and TBI groups (*p* < 0.01, *p* < 0.01). Within ECog scores, the PTSD/TBI_PTSD groups showed worse memory ability in contrast to healthy controls (*p* < 0.0001, *p* < 0.001), while the PTSD group showed worse visuospatial ability (*p* < 0.00001); planning and divided attention abilities were significantly worse in both groups compared to controls (*p* < 0.0001). MMSE and MoCA scores showed no significant differences between groups (*p* > 0.05). The AMEreading scores showed that the PTSD group had significantly more errors compared to controls (*p* < 0.001). Full scale IQ test score was significantly lower in the PTSD group compared to healthy controls (*p* < 0.01); see Table 1, Fig. 2.

**Table 1:** Demographics and Neuropsychological performance by groups.

	Healthy	TBI	TBI_PTSD	PTSD
	Mean (STD)	Mean (STD)	Mean (STD)	Mean (STD)
Number of subjects	57	21	31	57
Number of CSF	27	9	6	23
Age	70.9 (6.01)	67.9 (4.4)	68.7 (3.1)	67.8 (3.7)
Males (%)	100	100	100	100
ADAS-Cog	10.8 (4.58)	9.4 (3.6)	11 (5.2)	12.8 (3.9)
CAPS Current	2.22 (4.14)	7.36 (6.21)	39.08 (11.96)	56.64 (11.48)
CDR	0.05 (0.2)	0.33 (0.56)	0.47 (0.24)	0.21 (0.27)
CES	10.89 (10.2)	17 (10.35)	24.4 (10.12)	24.13 (8.88)
GDtotal	0.712 (0.91)	1.41 (1.91)	2.96 (2.6)	4 (2.82)
ECog memory	13.31 (5.25)	13.06 (5.47)	17.22 (5.77)	16.06 (5.67)
ECog language	13.12 (5.86)	12.53 (5.61)	15.93 (6.43)	17.11 (7.25)
ECog visspat	7.68 (1.24)	8.82 (3.52)	9.48 (3.98)	9.89 (3.72)
ECog plan	5.88 (1.5)	7.06 (3.54)	7.96 (3.19)	7.7 (2.76)
ECog divatt	5.66 (2.09)	6.53 (2.83)	8.22 (3.75)	7.85 (3.29)
ECog total	54.61 (15.08)	57.82 (22.97)	70.74 (23.72)	69.74 (23.35)
MMSE	28.75 (1.28)	28.94 (1.03)	28.22 (1.53)	28.09 (1.66)
MOCA	24.86 (2.76)	25.18 (3.36)	23.41 (2.89)	23.49 (3.84)
AMEreading	12.73 (8.11)	14.41 (8.43)	14.22 (10.77)	18.55 (11.27)
FSIQ	117.2 (6.65)	115.7 (6.880)	115.9 (8.93)	112.4 (9.28)

MoCA, Montreal Cognitive Assessment; ECog, Everyday Cognition; CAPS, Clinician-Administered PTSD Scale; MMSE, Mini-Mental State Exam; ADAS-COG, Alzheimer's Disease Assessment Scale-Cognitive; CDR, Clinical Dementia Rating; GDtotal, Geriatric Depression Scale; AMEreading, American Reading test; FSIQ, Full Scale Intelligence Quotient; CES, Center for Epidemiologic Studies Depression Scale.

### 3.2 Amyloid deposition in TBI and/or PTSD groups

**Fig. 3:**
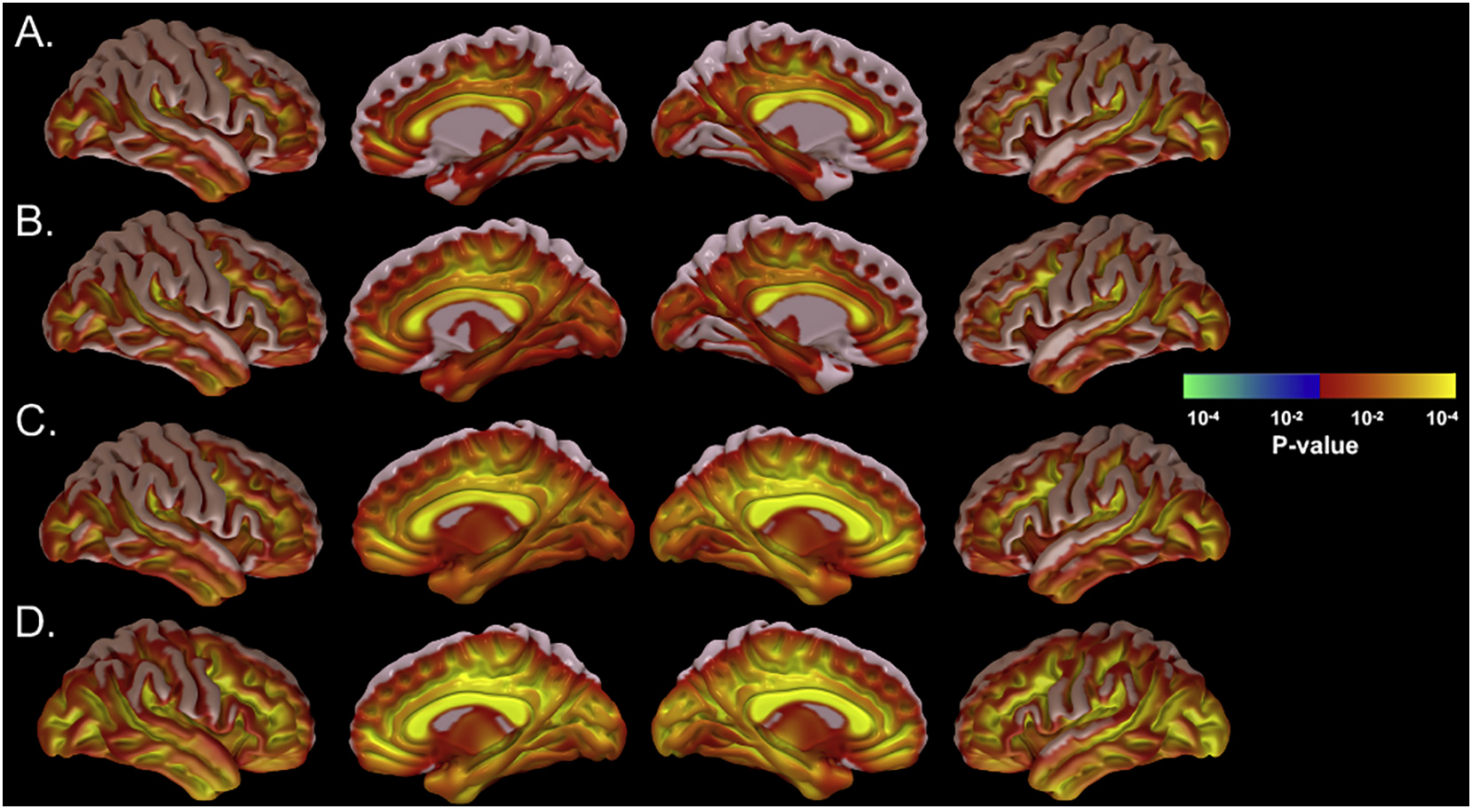
[^18^F]-AV45 SUVR average maps for each group showing region with amyloid positive uptake in the brain for different groups. Healthy controls showed the lowest uptake (A), while clinical groups showed more uptake in TBI, with higher in TBI_PTSD and the highest SUVR values in PTSD.

Fig. 3. illustrates the p-statistic maps of each group mean SUVR maps, revealing regions with significantly elevated uptake (SUVR >1.1) for each of the four study groups. There were sparse cortical regions of higher tracer uptake in the healthy control groups, but these regions were more extensive in the clinical groups. Interestingly, the TBI_PTSD group showed increased mean SUVR in the white matter while PTSD showed the highest zones of cortical SUVR relative to controls, see Fig. 3.

### 3.3 Group differences in amyloid deposition between TBI and/or PTSD groups

**Fig. 4:**
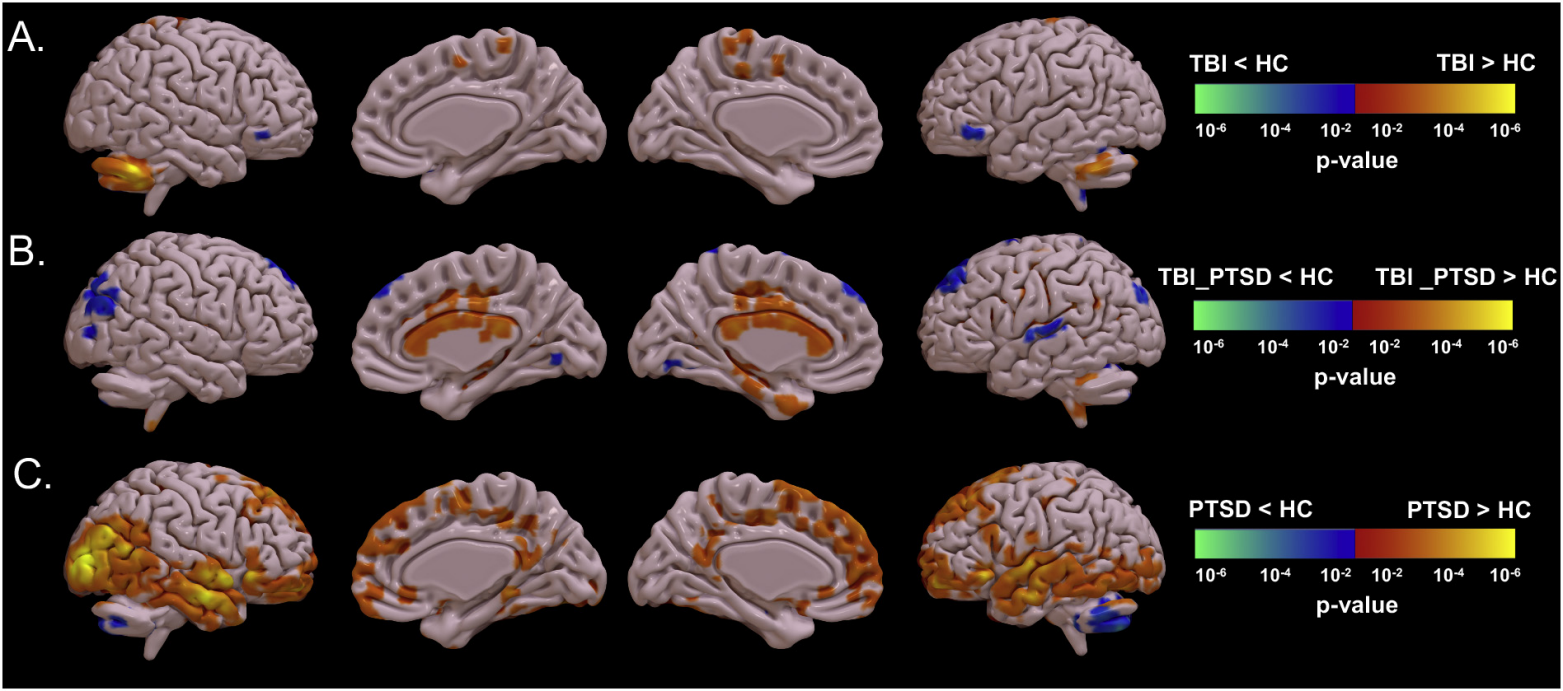
Groups comparisons of [^18^F]-AV45 SUVR between clinical groups and healthy group. A. TBI vs. healthy controls, B. TBI_PTSD vs. healthy control, and C. PTSD vs. healthy control. Red-yellow means higher means higher amyloid in clinical groups while blue-green means higher amyloid in healthy controls. Difference maps were calculated using unpaired *t*-test amyloid in clinical groups and blue and green test and results were corrected using cluster-based correction (*p* < 0.05, Z > 2.3, cluster >40 voxels). (For interpretation of the references to color in this figure legend, the reader is referred to the web version of this article.)

**Fig. 5:**
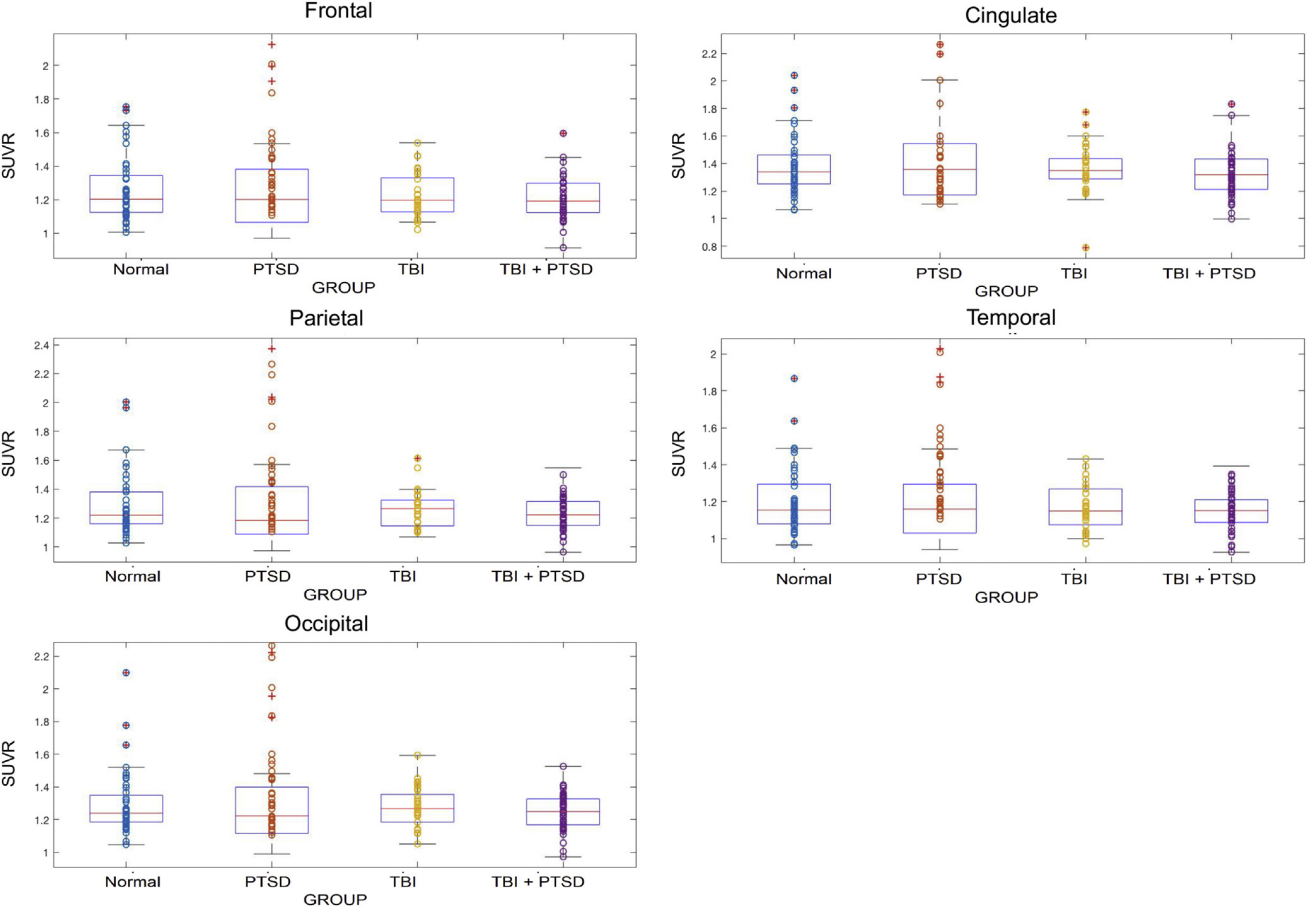
Shows the referenced standard uptake values (SUVR) for the five brain lobes. It shows significant SUVR differences between groups in the five VOIs, i.e. frontal, cingulate, parietal, occipital and temporal lobes.

Significant differences in [^18^F]-AV45 SUVR in the three clinical groups compared to the controls are represented in Fig. 4. TBI subjects had significantly higher [^18^F]-AV45 SUVR in the cerebellar tonsil (1.32 ± 0.21 versus 1.12 ± 0.25; *p* = 0.038) and precuneus (1.25 ± 0.21 versus 0.87 ± 0.20; *p* = 0.029) compared to controls, while SUVR was lower in ventrolateral prefrontal cortex (1.12 ± 0.2 versus1.2 ± 0.19; *p* = 0.041); see Fig. 4A and Fig 5.

The TBI_PTSD group showed increased [^18^F]-AV45 SUVR relative to controls, which was more localized to the white matter (1.6 ± 0.31 versus 1.32 ± 0.34; *p* = 0.027) especially in the corpus callosum (1.84 ± 0.5 versus 1.52 ± 0.5; *p* = 0.009), cingulate cortex (1.52 ± 0.36 versus 1.31 ± 0.4; *p* = 0.01), the middle temporal gyrus (1.25 ± 0.185 versus 1.12 ± 0.26; *p* = 0.048), while uptake was significantly lower comparted to controls in the middle frontal gyrus (1.21 ± 0.35 versus 1.48 ± 0.21; *p* = 0.032), superior temporal gyrus (1.5 ± 0.28 versus 1.7 ± 0.19; *p* = 0.028), and inferior parietal cortex (1.2 ± 0.21 versus 1.42 ± 0.34; *p* = 0.031); see Fig. 4B and Fig 5.

PTSD group had significantly higher [^18^F]-AV45 SUVR in various cortical areas including the prefrontal cortex (1.43 ± 0.2 versus 0.93 ± 0.41; *p* = 0.041), orbital gyrus (1.52 ± 0.29 versus 1.18 ± 0.21; *p* = 0.034), superior, middle and inferior temporal gyrus (1.2 ± 0.23 versus 1.21 ± 0.24; *p* = 0.032), hippocampus (1.56 ± 0.3 versus 1.3 ± 0.41; *p* = 0.031), middle and inferior occipital gyrus (1.52 ± 0.18 versus 1.3 ± 0.21; *p* = 0.021), posterior cingulate gyrus (1.2 ± 0.12 versus 0.92 ± 0.41; *p* = 0.05), and supplementary motor area (1.52 ± 0.32 versus 1.3 ± 0.31; *p* = 0.04), all compared to controls; see Fig. 4C and Fig 5.

### 3.4 Correlation between amyloid tracer uptake in brain and amyloid and tau levels in CSF

**Fig. 6:**
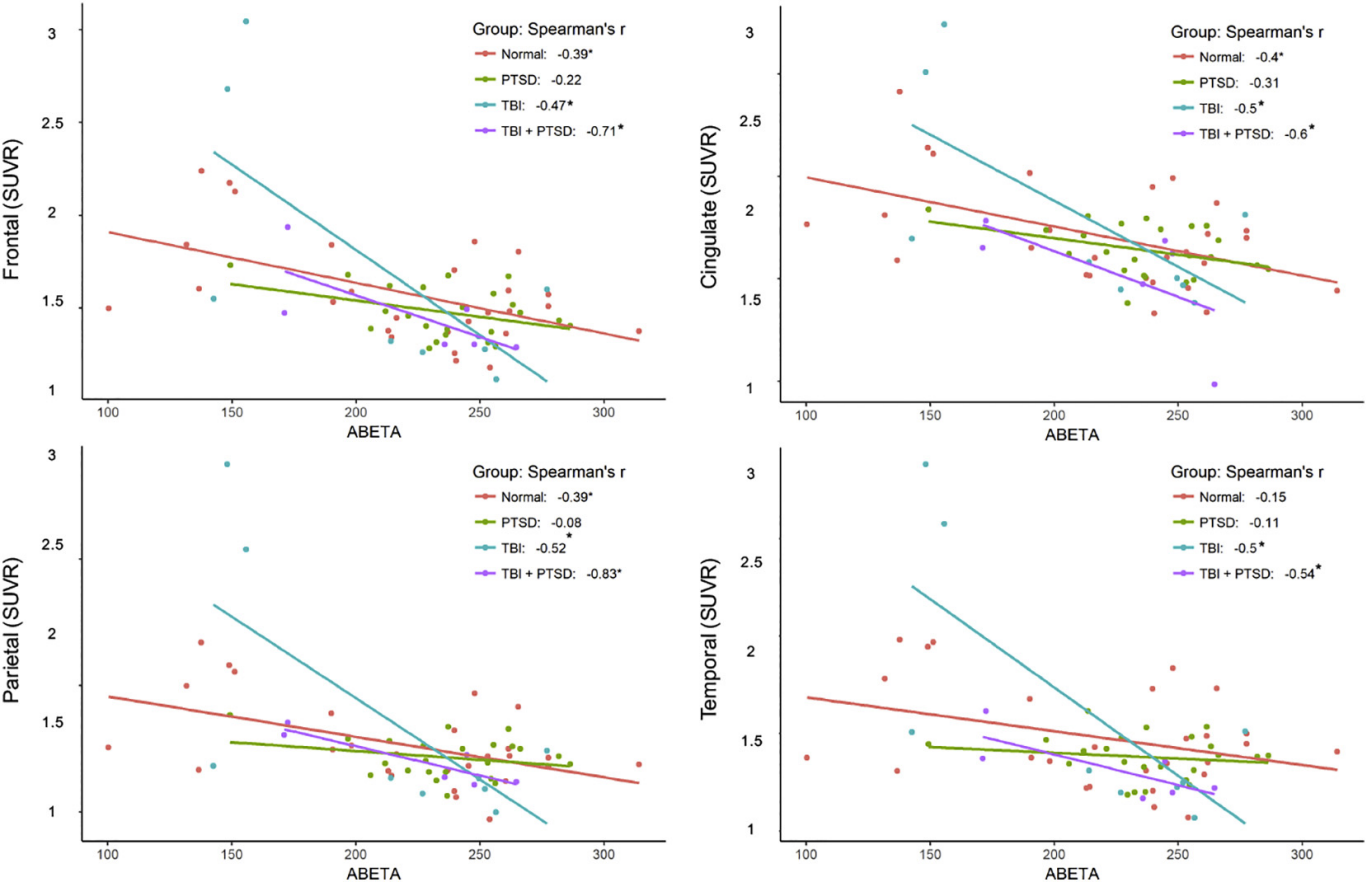
Shows the correlation between SUVR in different lobes of the brain and the amyloid in cerebrospinal fluid. There were significant negative correlations observed in both TBI and TBI_PTSD groups.

Fig. 6 shows the correlations between [^18^F]-AV45 SUVR in four ROIs with the CSF amyloid concentrations. Healthy controls showed negative correlation in frontal (r2 = 0.15; *p* = 0.046), parietal (r2 = 0.152; *p* = 0.045), and cingulate (r2 = 0.16; *p* = 0.036) cortices. Interestingly we observed significant negative correlations in the TBI-alone group in frontal (r2 = 0.22; *p* = 0.03), parietal (r2 = 0.27; *p* = 0.036), cingulate (r2 = 0.25; *p* = 0.04), and temporal cortex (r2 = 0.3; *p* = 0.033), and also in the TBI_PTSD group for frontal (r2 = 0.51; *p* = 0.026), parietal (r2 = 0.69; *p* = 0.03), cingulate (r2 = 0.36; *p* = 0.02), and temporal cortex (r2 = 0.13; *p* = 0.04); see Fig. 6 and Table 2.

**Table 2:** Correlations between amyloid in the brain and cerebrospinal fluid concentrations of amyloid and tau.

		Healthy	TBI	TBI_PTSD	PTSD
		R^2^ (*p*)	R^2^ (*p*)	R^2^ (*p*)	R^2^ (*p*)
CSF Amyloid	Frontal	0.15 (*p* = 0.046)	0.22 (*p* = 0.03)	0.51 (*p* = 0.026)	0.05 (*p* = 0.85)
	Parietal	0.152 (*p* = 0.046)	0.27 (*p* = 0.036)	0.69 (*p* = 0.03)	0 (*p* = 0.3)
	Cingulate	0.16 (*p* = 0.036)	0.25 (*p* = 0.04)	0.36 (*p* = 0.02)	0.09 (*p* = 0.67)
	Temporal	0.02 (*p* = 0.8)	0.3 (*p* = 0.033)	0.13 (*p* = 0.04)	0.01(*p* = 0.7)
	Frontal	0.16 (*p* = 0.27)	0.36 (*p* = 0.043)	0 (*p* = 0.9)	0.03 (*p* = 0.7)
CSF Tau	Parietal	0.08 (*p* = 0.12)	0.33 (*p* = 0.029)	0 (*p* = 0.86)	0 (*p* = 0.2)
	Cingulate	0.09 (*p* = 0.3)	0.3 (*p* = 0.036)	0.09 (*p* = 0.6)	0.12 (*p* = 0.4)
	Temporal	0.24 (*p* = 0.01)	0.3 (*p* = 0.01)	0.14 (*p* = 0.34)	0 (*p* = 0.3)

**Fig. 7:**
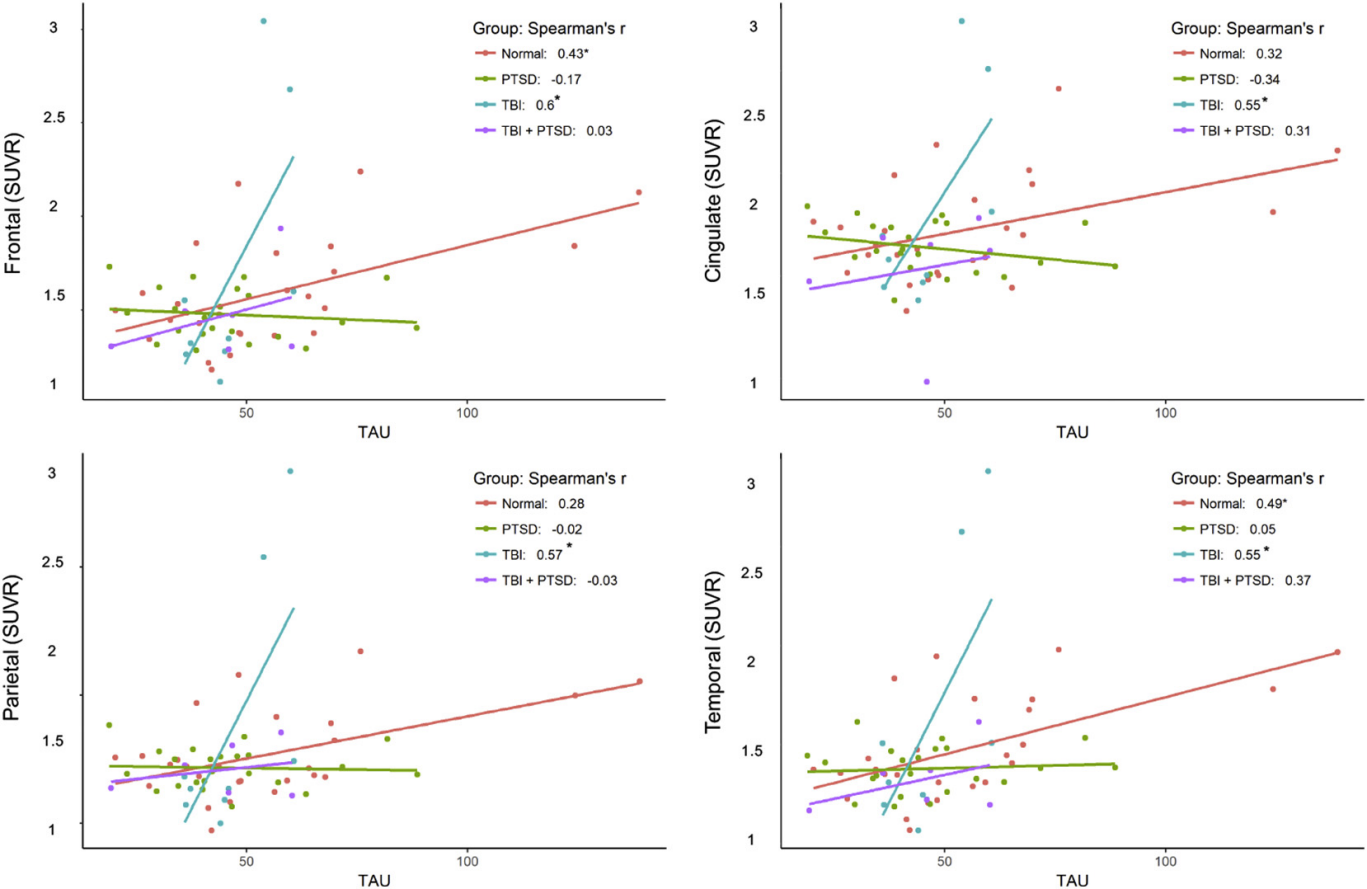
Shows the correlation between SUVR in different lobes of the brain and the tau in cerebrospinal fluid. Significant positive correlations observed in all regions for TBI and only in frontal lobe for TBI_PTSD.

Fig. 7 shows the correlations between individual [^18^F]-AV45 SUVR in four cortical ROIs with CSF tau-protein concentrations. Significant positive correlations were observed in the healthy control group within frontal (r2 = 0.16; *p* = 0.027) and temporal cortex (r2 = 0.24; *p* = 0.01). Significant positive correlations were observed in the TBI group within frontal (r2 = 0.36; *p* = 0.043), cingulate (r2 = 0.3; *p* = 0.036), parietal (r2 = 0.33; *p* = 0.029), and temporal cortex (r2 = 0.3; *p* = 0.01). There were no such significant correlations in the PTSD or TBI_PTSD groups; see Fig. 7 and Table 2.

## 4 Discussion

We used [^18^F]-AV45 PET imaging to investigate Aβ deposition in brain of Vietnam War veterans, seeking in particular to identify effects of PTSD alone or in association with TBI on this neuropathological marker. Earlier analysis of the data, considered the whole cerebral cortex as single ROI, had failed to reveal evidence for amyloidosis in brain of the veterans with PTSD and/or TBI (Weiner et al., 2017), but we felt that a voxel-wise statistical comparison might reveal regional patterns of amyloid accumulation not evident in that study. Indeed, we found notable accumulations of amyloid in the frontal, occipital and temporal lobes of PTSD sufferers with significant cognitive dysfunction, and in the white matter of TBI_PTSD group. Furthermore, we found correlation between amyloid PET results and CSF markers of neuropathology in the TBI and TBI_PTSD groups.

### 4.1 Neurocognition as a marker of progression to AD

A broad battery of neuropsychological tests had been collected to assess cognitive function in these Vietnam war veterans. The PTSD patients exhibited the worst cognitive performance on all assessments, followed by TBI_PTSD subjects, whereas cognitive scores in the TBI and control groups did not differ (Fig. 2).

TBI is a risk factor for PTSD (Xue et al., 2015), and both bring high risk for developing dementia later in life (Alway et al., 2016; Ruigrok et al., 2014). Indeed, 63% of our TBI participants had developed PTSD with a life CAPS score > 40 and current CAPS score > 30. This is consistent with previous findings that veterans with deployment-related TBI had more severe post-deployment PTSD symptoms (Yurgil et al., 2014), and that most TBI patients also suffer from PTSD (Yurgil et al., 2014). The causal link between TBI and PTSD may relate to the physical and cognitive impairment arising from the TBI, both of which increase PTSD prevalence (Koren et al., 2005; Xue et al., 2015). Alternately, emotional trauma arising in proportion to severity of the TBI may be the driver for developing PTSD (Yurgil et al., 2014).

We observed reduced cognitive function in both the PTSD and TBI_PTSD groups for all sub-categories of the ECog scale. While prior studies have not used ECog to investigate cognitive performance in relation to daily life of PTSD patients, several studies report lower cognitive function among PTSD patients (Cohen et al., 2013; Greenberg et al., 2014; Russman Block et al., 2017). The present changes suggest that altered brain structure or function following TBI may be associated with a risk for developing PTSD, neurodegenerative disease, or cognitive impairment.

In a recent study, Li et al. showed that age at the time of TBI influences ECog scores, with significantly higher ECog scores in all domains among patients experiencing their TBI during childhood (Li et al., 2017). We suppose that TBI in young adulthood may likewise have propagated to more severe cognitive effects in the present group of veterans. The mean GDtotal score was higher in PTSD and TBI_PTSD groups compared to TBI alone and control groups, consistent with the previous report (Marmar et al., 2015), which showed that 30% percent of PTSD patients had depression symptoms or depressive disorders 40 years after their Vietnam war service. The low incidence of behavioral and substance abuse disorders in our PTSD patients matches the findings of a previous study, where incidence of illicit drug use declined from 30% in 1972 to only 8% in 1996 (Balan et al., 2013).

### 4.2 Amyloid PET findings in the three clinical groups

TBI is now understood to provoke an acute increase of amyloid precursor protein (APP), the source for amyloid plaque formation, in response to axonal injury (Hefter and Draguhn, 2017). Diffusion tensor imaging (DTI) consistently shows white matter changes due to axonal injury and tract degradation post-TBI (Kou et al., 2010). The present TBI group showed clusters of increased Aβ deposition mainly in the cerebellum and precuneus regions (Fig. 4), rather matching the pattern of increased [^11^C]-PiB binding reported in TBI patients scanned 1–17 years after injury (Scott et al., 2016). Others reported Aβ plaque evident to PET and histopathology examination performed directly after the TBI (Ikonomovic et al., 2004; Roberts et al., 1994), and increased [^11^C]-PiB binding in cortex but not white matter of TBI patients scanned within one year of injury (Hong et al., 2014). However, presumed short term increases in plaque density were not evident to histopathological examination at some three years after the injury, despite an ongoing elevation of APP in the white matter (Chen et al., 2009). This implies that transient Aβ plaques can be substantially cleared in the years after TBI. In a post mortem study of long term TBI survivors, approximately one third had increased Aβ plaques, leading the authors to conclude that this subset of TBI survivors were at increased risk for developing AD (Johnson et al., 2012). We suppose that the large variance of [^18^F]-AV45 SUVR in our TBI group of only 18 subjects may conceal some individuals with more pronounced amyloid levels.

The TBI_PTSD group showed increased [^18^F]-AV45 SUVR throughout the white matter, most clearly evident in the corpus callosum (Fig. 4B). Indeed, amyloid PET tracers characteristically label the white matter, irrespective of amyloid content (Byun et al., 2017), although other studies reported a subtle increase in [^18^F]-AV45 SUVR in white matter of AD patients compared to healthy controls, (Nemmi et al., 2014). Therefore we cautiously attribute the present findings in the TBI_PTSD group to indicate Aβ accumulation in white matter. Indeed, there is concurrence of Aβ deposition and altered DTI in white matter of humans with TBI (Bendlin et al., 2012), and, a DTI study reported that white matter was more compromised in TBI_PTSD patients than in those with TBI alone (Lepage et al., 2017). Although not directly comparable to studies of recent injuries, we suppose that concurrent PTSD may have interfered with the recovery of axonal injury upon TBI in these veterans. In addition, we speculate that the history of TBI 40 years earlier initiated an Aβ pathology similar to that of AD, although it remains to be seen if this TBI cohort will progress to AD. To determine if the PET tracer used in this study could accurately predict the presence of β-amyloid in the brain at autopsy, we looked at the literature and found it does (Clark et al., 2011; Ikonomovic et al., 2016). In the study performed by Clark et al., 28 of a series of 29 AD patients who underwent Florbetapir-PET one year antimortem showed concord between PET and histopathological findings for β-amyloid positivity or negativity (Clark et al., 2011). In addition, Ikonomovic et al. showed that 90% of PET assessments of AD patients accurately reflected the amyloid plaque burden revealed from postmortem histology (Ikonomovic et al., 2016).

Veterans with PTSD have a two-fold increased risk for developing AD (Yaffe et al., 2010), but few studies have investigated the presence of Aβ in human PTSD. In animal models of PTSD entailing chronically elevated stress, the formation rate of amyloid plaques increased (Devi et al., 2010; Justice et al., 2015; Rothman et al., 2012). Amyloid plaque deposition in AD has been a long-standing topic. Several imaging studies have demonstrated that Aβ feature of the AD brain; Johnson et al compared 45 AD patients in contrast to 79 healthy controls and showed that the AD group showed higher [18F]-AV45 uptake in the precuneus, frontal, temporal, parietal, and anterior and posterior cingulate cortices (Johnson et al., 2013), with same pattern shown by Scott et al. (Scott et al., 2016), and supported by review reports (Chételat et al., 2013; Morris et al., 2016; Vlassenko et al., 2012). Interestingly, it has been shown that a history of PTSD symptoms increases the risk for developing AD (Qureshi et al., 2010; Yaffe et al., 2010b). Our results reflect similar findings where increased levels of Aβ are seen frontal, temporal, parietal, and anterior and posterior cingulate cortices in the brain of PTSD subjects that overlap with amyloid increase correspondent to AD (see Table 3). This may suggest that amyloid could be a possible link between PTSD and AD supported by the overlapping etiologies for PTSD and AD. One possible cause for amyloid presence could be the stress-related changes in the hypothalamic–pituitary–adrenal (HPA) axis (Morris et al., 2012). Indeed, PTSD and AD are both characterized by volumetric loss in the hippocampus in association with cognitive decline (Justice et al., 2015b; Tsolaki et al., 2009). The hippocampus is a particularly plastic and vulnerable region of the brain (McEwen, 1999). Several MRI studies of PTSD patients have shown decreased volumes of hippocampus (Lindauer et al., 2005; Schuff et al., 2008; van Rooij et al., 2015), anterior cingulate cortex (Schuff et al., 2008; Sekiguchi et al., 2013), and prefrontal structures (Woodward et al., 2009). Smaller hippocampal volumes have been repeatedly associated with development (Gilbertson et al., 2002) and persistence (van Rooij et al., 2015) of PTSD symptoms. In contrast, some studies showed no volumetric increases in hippocampus even after full recovery from PTSD symptoms (Lindauer et al., 2005; van Rooij et al., 2015). Thus, the causal relation between PTSD and volumetric changes in PTSD patients remains uncertain. In addition, environmental and genetic factors can contribute importantly to the risk of developing PTSD (Gilbertson et al., 2002; Lebois et al., 2016). In support of a HPA contribution to neurodegenerative disease progression, corticosteroid levels are elevated in early AD patients (Csernansky et al., 2006). In this work, we investigated effects of PTSD decades following the diagnosis, and establish a tentative link between PTSD and β-amyloid accumulation in the brain. However, diverse factors and mechanisms may have had a direct impact on our β-amyloid findings; PTSD and AD are both highly associated with increased neuro-inflammatory mediators, including cytokines, chemokines, and other neurotoxic mediators such as tryptase, histamine, IL-1β, TNF-α, IL-6, CCL2, IL-8, ROS, CRH, and MMPs, any of which might contribute to neurodegeneration (Kempuraj et al., 2017) and β-amyloid accumulation. Again, the cross-sectional design cannot establish the causal relationship between longer duration of PTSD and increased β-amyloid burden.

**Table 3:** Regions with increased β-amyloid with similar spatial distribution of β-amyloid both in TBI, PTSD and AD.

	TBI (Scott et al., 2016)	PTSD	AD (Scott et al., 2016)
Cerebellum	yes	Not reported	Less than TBI
Cingulate	Not reported	Reported	Reported
Pre-frontal Cortex	Not reported	Reported	Reported
Medial prefrontal cortex	Not reported	Reported	Reported
Temporal Cortex	Not reported	Reported	Reported
Parietal cortex	Not reported	Reported	Reported
Precuneus	Reported	Not reported	Reported
Occipital	Not Reported	Reported	Not reported

### 4.3 Correlation between SUVR in the brain and CSF amyloid and tau

The CSF concentration of Aβ is reduced while tau levels are elevated in AD patients (Apostolova et al., 2010; Toledo et al., 2013), and these typical CSF findings correlate with cerebral amyloid burden in PET (Masters and Selkoe, 2012; Scott et al., 2016). Although we found no significant correlations between our CSF markers and regional [^18^F]-AV45 SUVR in the PTSD and control groups, amyloid PET results in the TBI and TBI_PTSD groups showed negative correlations with CSF Aβ concentration and positive correlations with CSF tau concentration. Thus, the TBI findings resemble those reported earlier for AD patients (Apostolova et al., 2010; Toledo et al., 2013).

In severe TBI patients, increased CSF tau protein and decreased CSF Aβ levels were predictive for poor clinical outcomes (Koerte et al., 2015; Neselius et al., 2012; Ojo et al., 2016; Tsitsopoulos and Marklund, 2013), whereas Neselius et al. (2012) reported CSF p-tau increase in boxers to correlate positively with the duration after TBI. In addition, the tau concentration in brain interstitial fluid was more increased in cases of focal cortical injury than in patients with axonal injury (Marklund et al., 2009; Tsitsopoulos and Marklund, 2013). As such, present results are consistent with a persistence of elevated tau secretion into CSF persisting decades after TBI alone or in conjunction with PTSD.

Present correlations between PET-SUVR with CSF markers in the TBI and TBI_PTSD groups may suggest link between the brain β-amyloid and CSF amyloid accumulation, which resembles the hallmark pathology of AD, and may thus suggest an increased risk TBI and TBI_PTSD patients to develop AD later in life. In addition, a similar trend of negative correlations was observed in aged healthy controls, as shown in Fig. 6. However, the mechanisms causing the increased β-amyloid in the brain both in the TBI and PTSD groups could differ. As shown from the results, we observed increased β-amyloid in the TBI only group, which localized to the cerebellum and precuneus. However, the group with TBI and PTSD showed a substantial increase of β-amyloid burden in the white matter, whereas the PTSD alone group showed main increases of β-amyloid at the frontal, occipital and temporal cortices. These differences between the three clinical groups in the spatial distribution and maximal SUVR may explain the differing relationships between β-amyloid to PET and CSF concentrations of tau and β-amyloid in all three groups.

This study is subject to limitations arising in relation to preinjury functioning (Anderson et al., 2012) and socioeconomic status attained in civilian life. (Hoofien et al., 2002). Notwithstanding, our voxel wise reanalysis of the DOD-ADNI data reveals focally increased Aβ accumulation in brain of PTSD and TBI patients, suggesting a partial overlap with AD pathology, with increased Aβ in the occipital and temporal cortex in PTSD patients, whereas increases are restricted to the white matter in the TBI_PTSD cases. Irrespective of anatomic location, the increased Aβ burden was associated with lower cognitive performance in these Vietnam war veterans. The limited number of CSF samples available is yet another limitation in making conclusive statements about observed correlations with brain amyloid SUVR values. Despite the limited number of subjects in the TBI and TBI_PTSD groups, we were nonetheless able to see a significant correlation between PET and CSF concentrations of β-amyloid and Tau in both clinical TBI groups. Further analysis with larger samples size will be required to confirm the patterns. The cross-sectional, non-randomized study design also adds to these limitations and caution should be taken when making inferences on the correlations between CSF and brain β-amyloid.

## 5 Conclusion

In a voxel-wise comparison of the [^18^F]-AV45 PET data, we detected focally increased Aβ in cortex of the PTSD group and in white matter of the TBI plus PTSD group. Thus, we see the first evidence of increased Aβ in long-term PTSD sufferers, suggesting that cortical pathology contributes to their cognitive deficits. Our voxel-wise analysis of [^18^F]-AV45 revealed patterns of increased Aβ uptake that had not been discernible when examining the cerebral cortex as a single volume of interest.

## Conflict of interest

The authors declare no competing financial interests or conflict of interests.

## Author contributions

A.Z.M, F.N., and DOD-ADNI designed research; A.Z.M and DOD-ADNI performed research; A.Z.M, H.S., T.G., A.U., and C.N.H. analyzed data; A.Z.M., P.C., H.S, F.N. wrote the paper.

## References

[bb0005] Aisen P.S., Petersen R.C., Donohue M.C., Gamst A., Raman R., Thomas R.G., Walter S., Trojanowski J.Q., Shaw L.M., Beckett L.A., Jack C.R., Jagust W., Toga A.W., Saykin A.J., Morris J.C., Green R.C., Weiner M.W. (2010). Clinical core of the Alzheimer's disease neuroimaging initiative: progress and plans. Alzheimers Dement..

[bb0010] Alway Y., McKay A., Gould K.R., Johnston L., Ponsford J. (2016). Factors associated with posttraumatic stress disorder following moderate to severe traumatic brain injury: a prospective study. Depress. Anxiety.

[bb0015] Amen D.G., Raji C.A., Willeumier K., Taylor D., Tarzwell R., Newberg A., Henderson T.A. (2015). Functional neuroimaging distinguishes posttraumatic stress disorder from traumatic brain injury in focused and large community datasets. PLoS One.

[bb0020] Anderson V., Le Brocque R., Iselin G., Eren S., Dob R., Davern T.J., McKinlay L., Kenardy J. (2012). Adaptive ability, behavior and quality of life pre and posttraumatic brain injury in childhood. Disabil. Rehabil..

[bb0025] Andersson J.L.R., Jenkinson M., Smith S. (2007). Non-linear registration aka spatial normalisation. FMRIB technial report. In Pract..

[bb0030] Apostolova L.G., Hwang K.S., Andrawis J.P., Green A.E., Babakchanian S., Morra J.H., Cummings J.L., Toga A.W., Trojanowski J.Q., Shaw L.M., Jack C.R., Petersen R.C., Aisen P.S., Jagust W.J., Koeppe R.A., Mathis C.A., Weiner M.W., Thompson P.M. (2010). 3D PIB and CSF biomarker associations with hippocampal atrophy in ADNI subjects. Neurobiol. Aging.

[bb0035] Balan S., Widner G., Shroff M., van den Berk-Clark C., Scherrer J., Price R.K. (2013). Drug use disorders and post-traumatic stress disorder over 25 adult years: role of psychopathology in relational networks. Drug Alcohol Depend..

[bb0040] Bendlin B.B., Carlsson C.M., Johnson S.C., Zetterberg H., Blennow K., Willette A.a., Okonkwo O.C., Sodhi A., Ries M.L., Birdsill A.C., Alexander A.L., Rowley H.a., Puglielli L., Asthana S., Sager M.a. (2012). CSF T-tau/Aβ42 predicts white matter microstructure in healthy adults at risk for Alzheimer's disease. PLoS One.

[bb0045] Byun B.H., Kim B. Il, Park S.Y., Ko I.O., Lee K.C., Kim K.M., Kim Y.K., Lee J.-Y., Bu S.H., Kim J.H., Chi D.Y., Ha J.H., Lim S.M. (2017). Head-to-head comparison of 11C-PiB and ^18^F-FC119S for Aβ imaging in healthy subjects, mild cognitive impairment patients, and Alzheimer's disease patients. Medicine (Baltimore).

[bb0050] Chen X.-H.H., Johnson V.E., Uryu K., Trojanowski J.Q., Smith D.H. (2009). A lack of amyloid β plaques despite persistent accumulation of amyloid β in axons of long-term survivors of traumatic brain injury. Brain Pathol..

[bb0055] Chételat G., La Joie R., Villain N., Perrotin A., De La Sayette V., Eustache F., Vandenberghe R. (2013). Amyloid imaging in cognitively normal individuals, at-risk populations and preclinical Alzheimer's disease. NeuroImage Clin..

[bb0060] Clark C.M., Schneider J.A., Bedell B.J., Beach T.G., Bilker W.B., Mintun M.A., Pontecorvo M.J., Hefti F., Carpenter A.P., Flitter M.L., Krautkramer M.J., Kung H.F., Coleman R.E., Doraiswamy P.M., Fleisher A.S., Sabbagh M.N., Sadowsky C.H., Reiman P.E.M., Zehntner S.P., Skovronsky D.M. (2011). Use of florbetapir-PET for imaging β-amyloid pathology. JAMA, J. Am. Med. Assoc..

[bb0065] Cohen B.E., Neylan T.C., Yaffe K., Samuelson K.W., Li Y., Barnes D.E. (2013). Post-traumatic stress disorder and cognitive function. J. Clin. Psychiatry.

[bb0070] Csernansky J.G., Dong H., Fagan A.M., Wang L., Xiong C., Holtzman D.M., Morris J.C. (2006). Plasma cortisol and progression of dementia in subjects with Alzheimer-type dementia. Am. J. Psychiatry.

[bb0075] Devi L., Alldred M.J., Ginsberg S.D., Ohno M. (2010). Sex- and brain region-specific acceleration of β-amyloidogenesis following behavioral stress in a mouse model of Alzheimer's disease. Mol. Brain.

[bb0080] Farias S.T., Mungas D., Reed B.R., Cahn-Weiner D., Jagust W., Baynes K., Decarli C. (2008). The measurement of everyday cognition (ECog): scale development and psychometric properties. Neuropsychology.

[bb0085] Finfer S., Cohen J. (2000). Severe traumatic brain injury. Resuscitation.

[bb0090] Fischl B. (2012). FreeSurfer. NeuroImage.

[bb0095] Folstein M.F., Folstein S.E., McHugh P.R. (1975). “Mini-mental-state”, a practical method for grading the cognitive state of patients for the clinician. J. Psychiatr. Res..

[bb0100] Gilbertson M.W., Shenton M.E., Ciszewski A., Kasai K., Lasko N.B., Orr S.P., Pitman R.K. (2002). Smaller hippocampal volume predicts pathologic vulnerability to psychological trauma. Nat. Neurosci..

[bb0105] Grabner G., Janke A.L., Budge M.M., Smith D., Pruessner J., Collins D.L. (2006). Symmetric Atlasing and Model Based Segmentation: An Application to the Hippocampus in Older Adult.

[bb0110] Greenberg M.S., Tanev K., Marin M.F., Pitman R.K. (2014). Stress, PTSD, and dementia. Alzheimers Dement..

[bb0115] Hardy J. (2002). The amyloid hypothesis of Alzheimer's disease: progress and problems on the road to therapeutics. Science.

[bb0120] Hefter D., Draguhn A. (2017). APP as a protective factor in acute neuronal insults. Front. Mol. Neurosci..

[bb0125] Hong Y.T., Veenith T., Dewar D., Outtrim J.G., Mani V., Williams C., Pimlott S., Hutchinson P.J.A., Tavares A., Canales R., Mathis C.A., Klunk W.E., Aigbirhio F.I., Coles J.P., Baron J.-C., Pickard J.D., Fryer T.D., Stewart W., Menon D.K. (2014). Amyloid imaging with carbon 11-labeled Pittsburgh compound B for traumatic brain injury. JAMA Neurol..

[bb0130] Hoofien D., Vakil E., Gilboa A., Donovick P.J., Barak O. (2002). Comparison of the predictive power of socio-economic variables, severity of injury and age on long-term outcome of traumatic brain injury: sample-specific variables versus factors as predictors. Brain Inj..

[bb0135] Ikonomovic M.D., Uryu K., Abrahamson E.E., Ciallella J.R., Trojanowski J.Q., Lee V.M.Y., Clark R.S., Marion D.W., Wisniewski S.R., DeKosky S.T. (2004). Alzheimer's pathology in human temporal cortex surgically excised after severe brain injury. Exp. Neurol..

[bb0140] Ikonomovic M.D., Buckley C.J., Heurling K., Sherwin P., Jones P.A., Zanette M., Mathis C.A., Klunk W.E., Chakrabarty A., Ironside J., Ismail A., Smith C., Thal D.R., Beach T.G., Farrar G., Smith A.P.L. (2016). Post-mortem histopathology underlying β-amyloid PET imaging following flutemetamol F 18 injection. Acta Neuropathol. Commun..

[bb0145] Isamah N., Faison W., Payne M.E., MacFall J., Steffens D.C., Beyer J.L., Krishnan K.R., Taylor W.D. (2010). Variability in frontotemporal brain structure: the importance of recruitment of African Americans in neuroscience research. PLoS One.

[bb0150] Ito K., Hutmacher M.M., Corrigan B.W. (2012). Modeling of Functional Assessment Questionnaire (FAQ) as continuous bounded data from the ADNI database. J. Pharmacokinet. Pharmacodyn..

[bb0155] Jenkinson M., Bannister P., Brady M., Smith S. (2002). Improved optimization for the robust and accurate linear registration and motion correction of brain images. NeuroImage.

[bb0160] Jenkinson M., Beckmann C.F., Behrens T.E.J., Woolrich M.W., Smith S.M. (2012). FSL. NeuroImage.

[bb0165] Johnson V.E., Stewart W., Smith D.H. (2010). Traumatic brain injury and amyloid-β pathology: a link to Alzheimer's disease?. Nat. Rev. Neurosci..

[bb0170] Johnson V.E., Stewart W., Smith D.H. (2012). Widespread tau and amyloid-beta pathology many years after a single traumatic brain injury in humans. Brain Pathol..

[bb0175] Johnson K.A., Sperling R.A., Gidicsin C.M., Carmasin J.S., Maye J.E., Coleman R.E., Reiman E.M., Sabbagh M.N., Sadowsky C.H., Fleisher A.S., Murali Doraiswamy P., Carpenter A.P., Clark C.M., Joshi A.D., Lu M., Grundman M., Mintun M.A., Pontecorvo M.J., Skovronsky D.M., AV45-A11 Study Group, A.-A. Study (2013). Florbetapir (F18-AV-45) PET to assess amyloid burden in Alzheimer's disease dementia, mild cognitive impairment, and normal aging. Alzheimers Dement..

[bb0180] Justice N.J., Huang L., Tian J.-B., Cole A., Pruski M., Hunt A.J., Flores R., Zhu M.X., Arenkiel B.R., Zheng H. (2015). Post-traumatic stress disorder-like induction elevates β-amyloid levels, which directly activates corticotropin-releasing factor neurons to exacerbate stress responses. J. Neurosci..

[bb0185] Kempuraj D., Selvakumar G.P., Thangavel R., Ahmed M.E., Zaheer S., Raikwar S.P., Iyer S.S., Bhagavan S.M., Beladakere-Ramaswamy S., Zaheer A. (2017). Mast cell activation in brain injury, stress, and post-traumatic stress disorder and Alzheimer's disease pathogenesis. Front. Neurosci..

[bb0190] Kerbler G.M., Fripp J., Rowe C.C., Villemagne V.L., Salvado O., Rose S., Coulson E.J., Alzheimer's Disease Neuroimaging Initiative A.D.N. (2015). Basal forebrain atrophy correlates with amyloid β burden in Alzheimer's disease. NeuroImage. Clin..

[bb0195] Koerte I.K., Lin A.P., Willems A., Muehlmann M., Hufschmidt J., Coleman M.J., Green I., Liao H., Tate D.F., Wilde E.A., Pasternak O., Bouix S., Rathi Y., Bigler E.D., Stern R.A., Shenton M.E. (2015). A review of neuroimaging findings in repetitive brain trauma. Brain Pathol..

[bb0200] Koren D., Norman D., Cohen A., Berman J., Klein E.M. (2005). Increased PTSD risk with combat-related injury: a matched comparison study of injured and uninjured soldiers experiencing the same combat events. Am. J. Psychiatry.

[bb0205] Kou Z., Wu Z., Tong K.A., Holshouser B., Benson R.R., Hu J., Haacke E.M. (2010). The role of advanced MR imaging findings as biomarkers of traumatic brain injury. J. Head Trauma Rehabil..

[bb0210] Landau S.M., Mintun M.A., Joshi A.D., Koeppe R.A., Petersen R.C., Aisen P.S., Weiner M.W., Jagust W.J. (2012). Amyloid deposition, hypometabolism, and longitudinal cognitive decline. Ann. Neurol..

[bb0215] Lebois L.A.M., Wolff J.D., Ressler K.J. (2016). Neuroimaging genetic approaches to posttraumatic stress disorder. Exp. Neurol..

[bb0220] Lepage C., de Pierrefeu A., Koerte I.K., Coleman M.J., Pasternak O., Grant G., Marx C.E., Morey R.A., Flashman L.A., George M.S., McAllister T.W., Andaluz N., Shutter L., Coimbra R., Zafonte R.D., Stein M.B., Shenton M.E., Bouix S. (2017). White matter abnormalities in mild traumatic brain injury with and without post-traumatic stress disorder: a subject-specific diffusion tensor imaging study. Brain Imaging Behav..

[bb0225] Li W., Risacher S.L., McAllister T.W., Saykin A.J., Alzheimer's Disease Neuroimaging Initiative, A.D.N (2017). Age at injury is associated with the long-term cognitive outcome of traumatic brain injuries. Alzheimers Dement..

[bb0230] Lindauer R.J.L., Vlieger E.-J., Jalink M., Olff M., Carlier I.V.E., Majoie C.B.L.M., Den Heeten G.J., Gersons B.P.R. (2005). Effects of psychotherapy on hippocampal volume in out-patients with post-traumatic stress disorder: a MRI investigation. Psychol. Med..

[bb0235] Liu G., Allen B., Lopez O., Aizenstein H., Boudreau R., Newman A., Yaffe K., Kritchevsky S., Launer L., Satterfield S., Simonsick E., Rosano C. (2015). Racial differences in gray matter integrity by diffusion tensor in black and white octogenarians. Curr. Alzheimer Res..

[bb0240] Marklund N., Blennow K., Zetterberg H., Ronne-Engström E., Enblad P., Hillered L. (2009). Monitoring of brain interstitial total tau and beta amyloid proteins by microdialysis in patients with traumatic brain injury. J. Neurosurg..

[bb0245] Marmar C., Schlenger W., Henn-Haase C., Qian M., Purchia E., Li M., Corry N., Williams C.S., Ho C., Horesh D., Karstoft K., Shalev A., Kulka R.A. (2015). Course of posttraumatic stress disorder 40 years after the Vietnam war: findings from the National Vietnam Veterans Longitudinal Study. JAMA Psychiat..

[bb0250] Masters C.L., Selkoe D.J. (2012). Biochemistry of amyloid β-protein and amyloid deposits in Alzheimer disease. Cold Spring Harb. Perspect. Med..

[bb0255] McEwen B.S. (1999). Stress and hippocampal plasticity. Annu. Rev. Neurosci..

[bb0260] Morris J.C. (1993). The clinical dementia rating (CDR): current version and scoring rules. Neurology.

[bb0265] Morris M.C., Compas B.E., Garber J. (2012). Relations among posttraumatic stress disorder, comorbid major depression, and HPA function: a systematic review and meta-analysis. Clin. Psychol. Rev..

[bb0270] Morris E., Chalkidou A., Hammers A., Peacock J., Summers J., Keevil S. (2016). Diagnostic accuracy of ^18^F amyloid PET tracers for the diagnosis of Alzheimer's disease: a systematic review and meta-analysis. Eur. J. Nucl. Med. Mol. Imaging.

[bb0275] Nasreddine Z.S., Phillips N.A., Bedirian V., Charbonneau S., Whitehead V., Collin I., Cummings J.L., Chertkow H. (2005). The Montreal Cognitive Assessment, MoCA: a brief screening tool for mild cognitive impairment. J. Am. Geriatr. Soc..

[bb0280] Nemmi F., Saint-Aubert L., Adel D., Salabert A.-S., Pariente J., Barbeau E.J., Payoux P., Péran P. (2014). Insight on AV-45 binding in white and grey matter from histogram analysis: a study on early Alzheimer's disease patients and healthy subjects. Eur. J. Nucl. Med. Mol. Imaging.

[bb0285] Neselius S., Brisby H., Theodorsson A., Blennow K., Zetterberg H., Marcusson J. (2012). CSF-biomarkers in olympic boxing: diagnosis and effects of repetitive head trauma. PLoS One.

[bb0290] Ojo J.O., Mouzon B.C., Crawford F. (2016). Repetitive head trauma, chronic traumatic encephalopathy and tau: challenges in translating from mice to men. Exp. Neurol..

[bb0295] Qureshi S.U., Kimbrell T., Pyne J.M., Magruder K.M., Hudson T.J., Petersen N.J., Yu H.J., Schulz P.E., Kunik M.E. (2010). Greater prevalence and incidence of dementia in older veterans with posttraumatic stress disorder. J. Am. Geriatr. Soc..

[bb0300] Roberts G.W., Gentleman S.M., Lynch A., Murray L., Landon M., Graham D.I. (1994). Beta amyloid protein deposition in the brain after severe head injury: implications for the pathogenesis of Alzheimer's disease. J. Neurol. Neurosurg. Psychiatry.

[bb0305] van Rooij S.J.H., Kennis M., Sjouwerman R., van den Heuvel M.P., Kahn R.S., Geuze E. (2015). Smaller hippocampal volume as a vulnerability factor for the persistence of post-traumatic stress disorder. Psychol. Med..

[bb0310] Rothman S.M., Herdener N., Camandola S., Texel S.J., Mughal M.R., Cong W.N., Martin B., Mattson M.P. (2012). 3xTgAD mice exhibit altered behavior and elevated Aβ after chronic mild social stress. Neurobiol. Aging.

[bb0315] Ruigrok A.N.V., Salimi-Khorshidi G., Lai M.-C., Baron-Cohen S., Lombardo M.V., Tait R.J., Suckling J. (2014). A meta-analysis of sex differences in human brain structure. Neurosci. Biobehav. Rev..

[bb0320] Russman Block S., King A.P., Sripada R.K., Weissman D.H., Welsh R., Liberzon I. (2017). Behavioral and neural correlates of disrupted orienting attention in posttraumatic stress disorder. Cogn. Affect. Behav. Neurosci..

[bb0325] Saint-Aubert L., Barbeau E.J., Péran P., Nemmi F., Vervueren C., Mirabel H., Payoux P., Hitzel A., Bonneville F., Gramada R., Tafani M., Vincent C., Puel M., Dechaumont S., Chollet F., Pariente J. (2013). Cortical florbetapir-PET amyloid load in prodromal Alzheimer's disease patients. EJNMMI Res..

[bb0330] Schuff N., Neylan T.C., Fox-Bosetti S., Lenoci M., Samuelson K.W., Studholme C., Kornak J., Marmar C.R., Weiner M.W. (2008). Abnormal N-acetylaspartate in hippocampus and anterior cingulate in posttraumatic stress disorder. Psychiatry Res. Neuroimaging.

[bb0335] Scott G., Ramlackhansingh A.F., Edison P., Hellyer P., Cole J., Veronese M., Leech R., Greenwood R.J., Turkheimer F.E., Gentleman S.M., Heckemann R.A., Matthews P.M., Brooks D.J., Sharp D.J. (2016). Amyloid pathology and axonal injury after brain trauma. Neurology.

[bb0340] Sekiguchi A., Sugiura M., Taki Y., Kotozaki Y., Nouchi T., Takeuchi H., Araki T., Hanawa S., Nakagawa S., Miyauchi C.M., Sakuma A., Kawashima R. (2013). Brain structural changes as vulnerability factors and acquired signs of post-earthquake stress. Mol. Psychiatry.

[bb0345] Skinner J., Carvalho J.O., Potter G.G., Thames A., Zelinski E., Crane P.K., Gibbons L.E. (2012). The Alzheimer's disease assessment scale-cognitive-plus (ADAS-Cog-Plus): an expansion of the ADAS-Cog to improve responsiveness in MCI. Brain Imaging Behav..

[bb0350] Spreen O., Strauss E. (1998). National Adult Reading Test (NART). A Compendium of Neuropsychological Tests.

[bb0355] Toledo J.B., Korff A., Shaw L.M., Trojanowski J.Q., Zhang J. (2013). CSF α-synuclein improves diagnostic and prognostic performance of CSF tau and Aβ in Alzheimer's disease. Acta Neuropathol..

[bb0360] Tsitsopoulos P.P., Marklund N. (2013). Amyloid-β peptides and tau protein as biomarkers in cerebrospinal and interstitial fluid following traumatic brain injury: a review of experimental and clinical studies. Front. Neurol..

[bb0365] Tsolaki M., Eleftheriou M., Karavida N. (2009). Alzheimer's dementia and post-traumatic stress disorder differences and similarities in neuroimaging. Hell. J. Nucl. Med..

[bb0370] Uryu K., Chen X.H., Martinez D., Browne K.D., Johnson V.E., Graham D.I., Lee V.M.Y., Trojanowski J.Q., Smith D.H. (2007). Multiple proteins implicated in neurodegenerative diseases accumulate in axons after brain trauma in humans. Exp. Neurol..

[bb0375] Vlassenko A.G., Benzinger T.L.S., Morris J.C. (2012). PET amyloid-beta imaging in preclinical Alzheimer's disease. Biochim. Biophys. Acta Mol. basis Dis..

[bb0380] Wang Z., Neylan T.C., Mueller S.G., Lenoci M., Truran D., Marmar C.R., Weiner M.W., Schuff N. (2010). Magnetic resonance imaging of hippocampal subfields in posttraumatic stress disorder. Arch. Gen. Psychiatry.

[bb0385] Weiner M.W., Harvey D., Hayes J., Landau S.M., Aisen P.S., Petersen R.C., Tosun D., Veitch D.P., Jack C.R., Decarli C., Saykin A.J., Grafman J., Neylan T.C. (2017). Effects of traumatic brain injury and posttraumatic stress disorder on development of Alzheimer's disease in Vietnam veterans using the Alzheimer's disease neuroimaging initiative: preliminary report. Alzheimer's Dement. Transl. Res. Clin. Interv..

[bb0390] Winkler A.M., Ridgway G.R., Webster M.A., Smith S.M., Nichols T.E. (2014). Permutation inference for the general linear model. NeuroImage.

[bb0395] Woodward S.H., Schaer M., Kaloupek D.G., Cediel L., Eliez S. (2009). Smaller global and regional cortical volume in combat-related posttraumatic stress disorder. Arch. Gen. Psychiatry.

[bb0400] Xue C., Ge Y., Tang B., Liu Y., Kang P., Wang M., Zhang L. (2015). A meta-analysis of risk factors for combat-related PTSD among military personnel and veterans. PLoS One.

[bb0405] Yaffe K., Vittinghoff E., Lindquist K., Barnes D., Covinsky K.E., Neylan T., Kluse M., Marmar C. (2010). Posttraumatic stress disorder and risk of dementia among US veterans. Arch. Gen. Psychiatry.

[bb0410] Yurgil K.A., Barkauskas D.A., Vasterling J.J., Nievergelt C.M., Larson G.E., Schork N.J., Litz B.T., Nash W.P., Baker D.G. (2014). Association between traumatic brain injury and risk of posttraumatic stress disorder in active-duty marines. JAMA Psychiat..

