# Corrigendum to ‘Amyloid pathology fingerprint differentiates post-traumatic stress disorder and traumatic brain injury’ NeuroImage: CLINICAL. 19 (2018) 716–726

**DOI:** 10.1016/j.nicl.2019.101829

**Published:** 2019-04-27

**Authors:** Abdalla Z. Mohamed, Paul Cumming, Hussein Srour, Tamara Gunasena, Aya Uchida, Courtney Nicole Haller, Fatima Nasrallah, for the Department of Defense Alzheimer's Disease Neuroimaging Initiative

**Affiliations:** a Queensland Brain Institute, The University of Queensland, Brisbane, QLD 4072, Australia; b School of Psychology and Counselling and IHBI, Queensland University of Technology, Brisbane, QLD 4059, Australia; c QIMR-Berghofer Institute, Brisbane, QLD 4006, Australia; d School of Medicine, The University of Queensland, Brisbane, QLD 4072, Australia

## 1 Introduction

The authors regret to inform that a few issues were found in the published paper and therefore, we wish to submit this Corrigenda to the original article entitled ‘Amyloid pathology fingerprint differentiates post-traumatic stress disorder and traumatic brain injury’ NeuroImage: Clinical. 19 (2018) 716–726.

## 2 Methods

The following text should be added to the first paragraph of Section 2.2 statistical analysis after this sentence “Neuropsychological data were analyzed using RStudio 3.3.3.”:

“Participants responded to the ECog questionnaire with a four-point scale (i.e. 1, 2, 3, or 4), or responded with 9 for “I don't know”, which we considered as missing information. To accommodate for such missing information, we performed “Missing at Random corrections” depending on the type of missing data.”

## 3 Results

The total number of subjects used in this article is 164, not 166. In Table 1, the number of subjects for whom neuropsychological data was available at the time of writing differed from the number of subjects with neuroimaging. Thus, Table 1 should read:

Table 1 Demographics and Neuropsychological performance by groups.

**Table:** 

	Healthy	TBI	TBI_PTSD	PTSD
Number of subjects in PET Analysis	57	21	29	57
Number of subjects with neuropsychological test scores reported	57	17	27	53
Number of subjects with CSF analysis	27	9	6	23
Age	70.9 (6.01)	67.9 (4.4)	68.7 (3.1)	67.8 (3.7)
Males (%)	100	100	100	100
MCI	2	2	3	5
ADAS-Cog	10.8 (4.58)	9.4 (3.6)	11 (5.2)	12.8 (3.9)⁎⁎
CAPS Current	2.22 (4.14)	7.36 (6.21)⁎⁎	39.08 (11.96)⁎⁎⁎⁎	56.64 (11.48)⁎⁎⁎⁎
CDR	0.05 (p = 0.2)	0.33 (p = 0.56)	0.47 (p = 0.24)⁎⁎	0.21 (p = 0.27)⁎⁎⁎
CES	10.89 (10.2)	17 (10.35)	24.4 (10.12)⁎⁎⁎⁎	24.13 (8.88)⁎⁎⁎⁎
GDtotal	0.712 (p = 0.91)	1.41 (1.91)	2.96 (2.6)⁎⁎⁎	4 (2.82)⁎⁎⁎⁎
ECog memory	13.31 (5.25)	13.06 (5.47)	17.22 (5.77)⁎⁎⁎	16.06 (5.67)⁎⁎⁎
ECog language	13.12 (5.86)	12.53 (5.61)	15.93 (6.43)⁎⁎⁎⁎	17.11 (7.25)⁎⁎⁎⁎
ECog visspat	7.68 (1.24)	8.82 (3.52)	9.48 (3.98)⁎⁎⁎⁎	9.89 (3.72)⁎⁎⁎
ECog plan	5.88 (1.5)	7.06 (3.54)⁎⁎	7.96 (3.19)⁎⁎⁎⁎	7.7 (2.76)⁎⁎⁎⁎
ECog divatt	5.66 (2.09)	6.53 (2.83)	8.22 (3.75)⁎⁎⁎	7.85 (3.29)⁎⁎⁎⁎
ECog total	54.61 (15.08)	57.82 (22.97)⁎⁎⁎	70.74 (23.72)⁎⁎⁎	69.74 (23.35)⁎⁎⁎⁎
MMSE	28.75 (1.28)	28.94 (1.03)	28.22 (1.53)	28.09 (1.66)
MOCA	24.86 (2.76)	25.18 (3.36)	23.41 (2.89)	23.49 (3.84)
AMEreading	12.73 (8.11)	14.41 (8.43)	14.22 (10.77)	18.55 (11.27)
FSIQ	117.2 (6.65)	115.7 (6.88)	115.9 (8.93)⁎	112.4 (9.28)⁎⁎

* p <0.05.

** p < 0.01.

*** p < 0.001.

**** p < 0.0001.

Section 3.3 should have presented a separate section for the voxelwise results in Fig. 4 and a separate section for the a priori-defined lobar regions of interest in Fig. 5. The following text should therefore replace the text in 3.3:

### 3.1 Group differences in amyloid deposition between TBI and/or PTSD groups

Significant differences in [^18^F]-AV45 SUVR in the three clinical groups compared to the controls are represented in Fig. 4. TBI subjects had significantly higher [^18^F]-AV45 SUVR in the cerebellar tonsil (1.32 ± 0.21 versus 1.12 ± 0.25; p = 0.038; MNI-coordinates −43.5, −56.5, −37), SMA (1.25 ± 0.21 versus 0.87 ± 0.20; p = 0.029; −3, −23.5, 65) and precuneus (1.23 ± 0.2 versus 0.91 ± 0.36; p = 0.04, −11, −56, 48.5) compared to controls, while SUVR was lower in ventrolateral prefrontal cortex (1.12 ± 0.2 versus 1.2 ± 0.19; p = 0.041; −54.5, 32, −5.5); see Fig. 4A.

The TBI_PTSD group showed increased [^18^F]-AV45 SUVR relative to controls, which was mainly localized to the white matter (1.60 ± 0.31 versus 1.32 ± 0.34; p = 0.027; 26, 8, 24.5), especially in the corpus callosum (1.84 ± 0.50 versus 1.52 ± 0.50; p = 0.009; 0.5, −32.5, 21.5), but also in the cingulate cortex (1.52 ± 0.36 versus 1.31 ± 0.40; p = 0.01; −7, 2, 39.5), the left medial temporal gyrus (1.25 ± 0.19 versus 1.12 ± 0.26; p = 0.048; −24, −19, 25), while uptake was significantly lower comparted to controls in the superior frontal gyrus (1.21 ± 0.35 versus 1.48 ± 0.21; p = 0.032; −8, 50, 48), superior temporal gyrus (1.50 ± 0.28 versus 1.7 ± 0.19; p = 0.028; −66.5,-13, 5), and inferior parietal cortex (1.20 ± 0.21 versus 1.42 ± 0.34; p = 0.031; 55, −70, 33.5); see Fig. 4B.

The PTSD group had significantly higher [^18^F]-AV45 SUVR in various cortical areas including the prefrontal cortex (1.43 ± 0.20 versus 0.93 ± 0.41; p = 0.041; −26, 33.5,47), orbital gyrus (1.52 ± 0.29 versus 1.18 ± 0.21; p = 0.034; −50,26,-7), superior, middle and inferior temporal gyrus (1.20 ± 0.23 versus 1.21 ± 0.24; p = 0.032; 61, 6.5, −10), middle and inferior occipital gyrus (1.52 ± 0.18 versus 1.30 ± 0.21; p = 0.021; 38.5,-89.5,3.5), posterior cingulate gyrus (1.20 ± 0.12 versus 0.92 ± 0.41; p = 0.05, 1,-49,33.5), and supplementary motor area (1.52 ± 0.32 versus 1.30 ± 0.31; p = 0.04; 1, 2, 53), with lower [^18^F]-AV45 SUVR in cerebellum (1.00 ± 0.21 versus 1.12 ± 0.25; p = 0.04; −42.0, −56.5, −37), all compared to controls; see Fig. 4C.

In contrast to these voxelwise analyses, lobar regions of interest analyses showed no significant group differences in the frontal, temporal, parietal, cingulate and occipital lobes (Fig. 5). For a comparative illustration of the voxelwise analysis results with these lobar regions of interest, we chose two clusters from the voxelwise map above where SUVR in the PTSD group exceeds that in the control group at a threshold 0.01 < p < 0.0001. The clusters were defined according to the SUVR differences between PTSD and normal groups in the bilateral temporal lobe. After applying the masks of left and right clusters to extract the average SUVR values for each individual, we then estimated the effect size for each comparison, as shown in the table below. Group differences for the contrast of PTSD versus control groups were of small effect size. According to the uncorrected (for multiple comparisons) difference maps we also saw small clusters showing differences between the TBI and normal groups, but these clusters did not survive correction for multiple comparison.

**Table:** 

Cluster of interest	PTSD vs. HC	TBI_PTSD vs. HC	TBI vs. HC
Right temporal cortex (6169 voxels; 20.8 cm^3^) MNI: 61, 6.5, −10	0.290	0.042	0.092
Left temporal cortex (5829 voxels; 19.7 cm^3^) MNI: −59, 5, −8	0.174	0.096	0.106

The following Fig. 5 should replace the original Fig. 5 in the manuscript.

**Figure:**
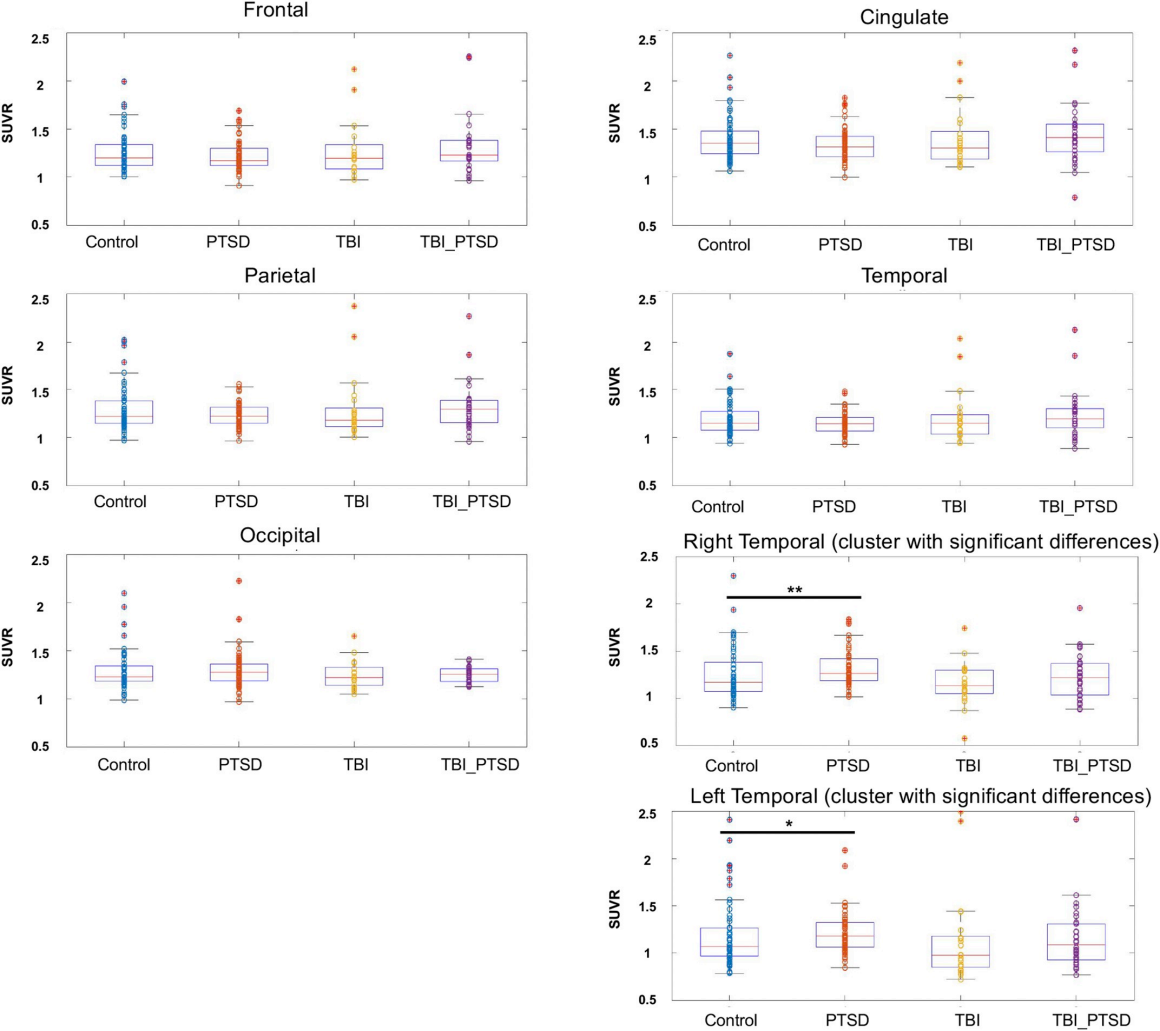

Fig. 5 The mean SUVR values in the frontal, cingulate, parietal, temporal, and occipital lobar regions of interest for the control (N = 57), PTSD (N = 57), TBI (N = 21), and TBI_PTSD (n = 29) groups. There were no significant group differences for any brain lobar region of interest (p > 0.05). The voxel cluster in the right temporal lobe with significant differences between the healthy and PTSD groups from the voxel wise analysis (i.e. results from Fig. 4C) had higher mean SUVR in the PTSD relative to the control group. The voxel cluster in the left temporal lobe had higher mean SUVR in the PTSD group than in the control group. Consistent with these scatter plots, Cohen's d effect sizes were small (0.29 in right temporal cortex, 0.17 in left temporal cortex).

We had inadvertently carried over the p-values in Table 2 from the earlier Pearson's r-correlation tests between SUVR by lobe and CSF levels of amyloid and tau. The following Table 2 should replace the Table 2 in the paper. The more stringent Spearman rho's p-values still show significant correlations between SUVR in the frontal and parietal lobes with CSF amyloid levels.

Table 2 Correlations between amyloid in the brain and cerebrospinal fluid concentrations of amyloid and tau.

**Table:** 

		Normal R^2^ (p)	TBI R^2^ (p)	TBI_PTSD R^2^ (p)	PTSD R^2^ (p)
CSF Amyloid	Frontal	0.15 (p = 0.05)*	0.22 (p = 0.21)	0.51 (p = 0.11)	0.05 (p = 0.32)
	Parietal	0.15 (p = 0.05)*	0.27 (p = 0.15)	0.69 (p = 0.04)*	0.01 (p = 0.71)
	Cingulate	0.16 (p = 0.04)*	0.25 (p = 0.17)	0.36 (p = 0.21)	0.10 (p = 0.15)
	Temporal	0.02 (p = 0.45)	0.25 (p = 0.17)	0.29 (p = 0.27)	0.01 (p = 0.61)
CSF Tau	Frontal	0.19 (p = 0.03)*	0.36 (p = 0.09)	0.0 (p = 0.96)	0.03 (p = 0.44)
	Parietal	0.08 (p = 0.17)	0.32 (p = 0.11)	0.0 (p = 0.96)	0.00 (p = 0.92)
	Cingulate	0.10 (p = 0.11)	0.30 (p = 0.13)	0.10 (p = 0.54)	0.12 (p = 0.11)
	Temporal	0.24 (p = 0.01)*	0.30 (p = 0.13)	0.14 (p = 0.47)	0.00 (p = 0.81)

Also propagating from our earlier use of Pearson's correlations in Table 2, we incorrectly reported certain correlations in Figs. 6 and 7 as being significant (*). The Spearman correlations between SUVR with CSF beta-amyloid concentration were significant in 6/16 of the cells in the table (Table 2; p < 0.05), as opposed to Pearson's correlations, which were significant in 11/16 of the cells in the table (Fig. 6). The Spearman correlations between SUVR with CSF tau concentration, were significant in 2/16 cells in the table (Table 2; p < 0.05), as opposed to Pearson's correlations, which were significant in 6/16 cells in the table (Fig. 7).

### 3.2 Correlation between amyloid tracer uptake in brain and amyloid and tau levels in CSF

Fig. 6 shows the correlations between [^18^F]-AV45 SUVR in four cortical lobe ROIs with the CSF amyloid concentrations. Healthy controls showed negative correlation in frontal (r^2^ = 0.15; p = 0.046), parietal (r^2^ = 0.15; p = 0.045), and cingulate (r^2^ = 0.16; p = 0.037) lobes. Interestingly we observed significant negative correlations in the TBI_PTSD group for parietal (r^2^ = 0.69; p = 0.042) lobes, with no significant correlations observed in the PTSD or TBI group (p > 0.05); see Fig. 6 and Table 2.

Fig. 7 shows the correlations between individual [^18^F]-AV45 SUVR in four cortical lobe ROIs with CSF tau-protein concentrations. Significant positive correlations were observed in the healthy control group in the frontal lobe (r^2^ = 0.19; p = 0.028). There were no such significant correlations in the TBI, PTSD or TBI_PTSD groups; see Fig. 7 and Table 2.

The following Fig. 6 and 7 should replace the original Fig. 6 and 7 in the manuscript:

**Figure:**
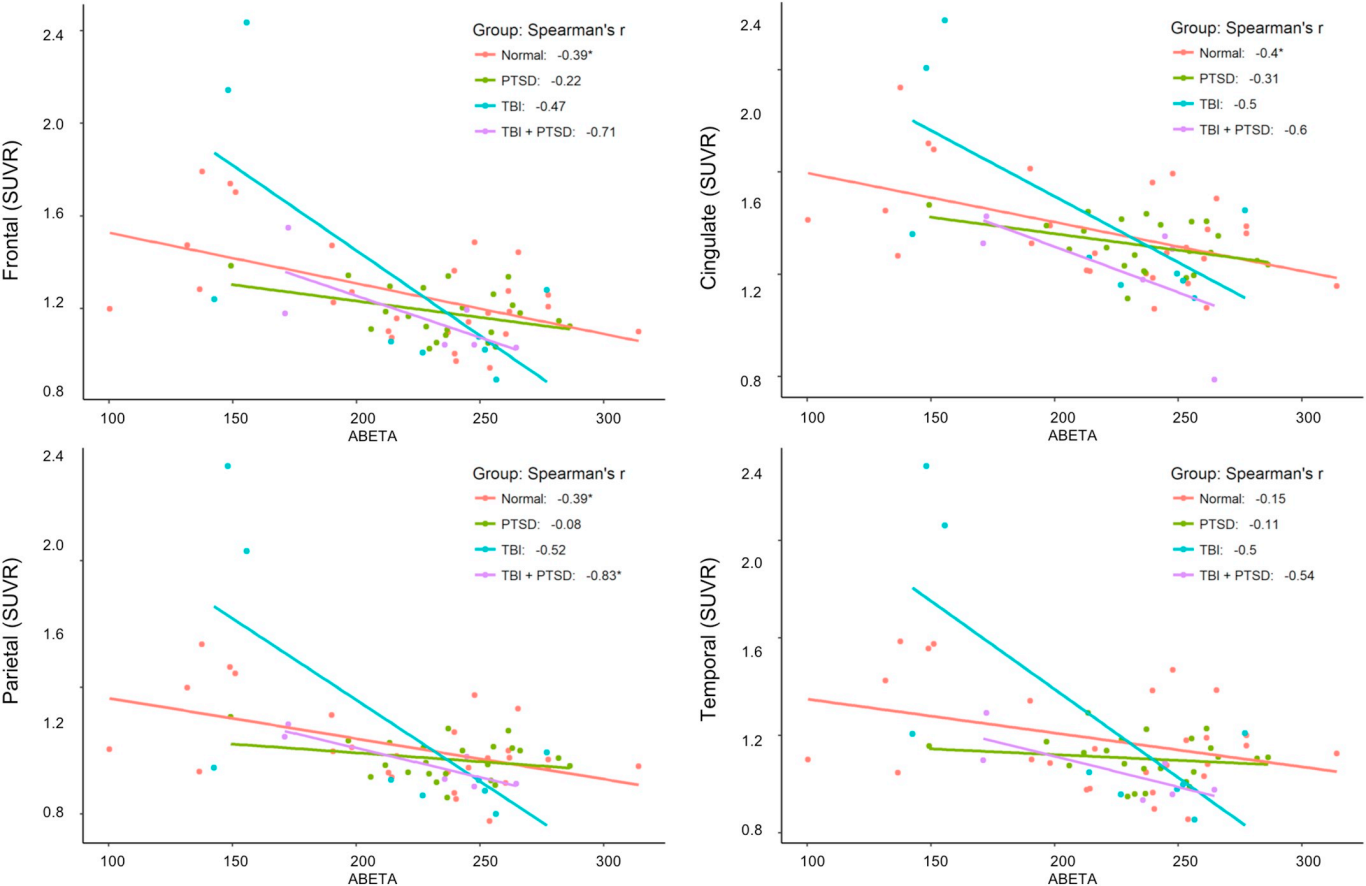

Fig. 6 shows the correlation between SUVR in different lobes of the brain and the amyloid concentration in cerebrospinal fluid. There were significant negative correlations observed in both TBI and TBI_PTSD groups.

**Figure:**
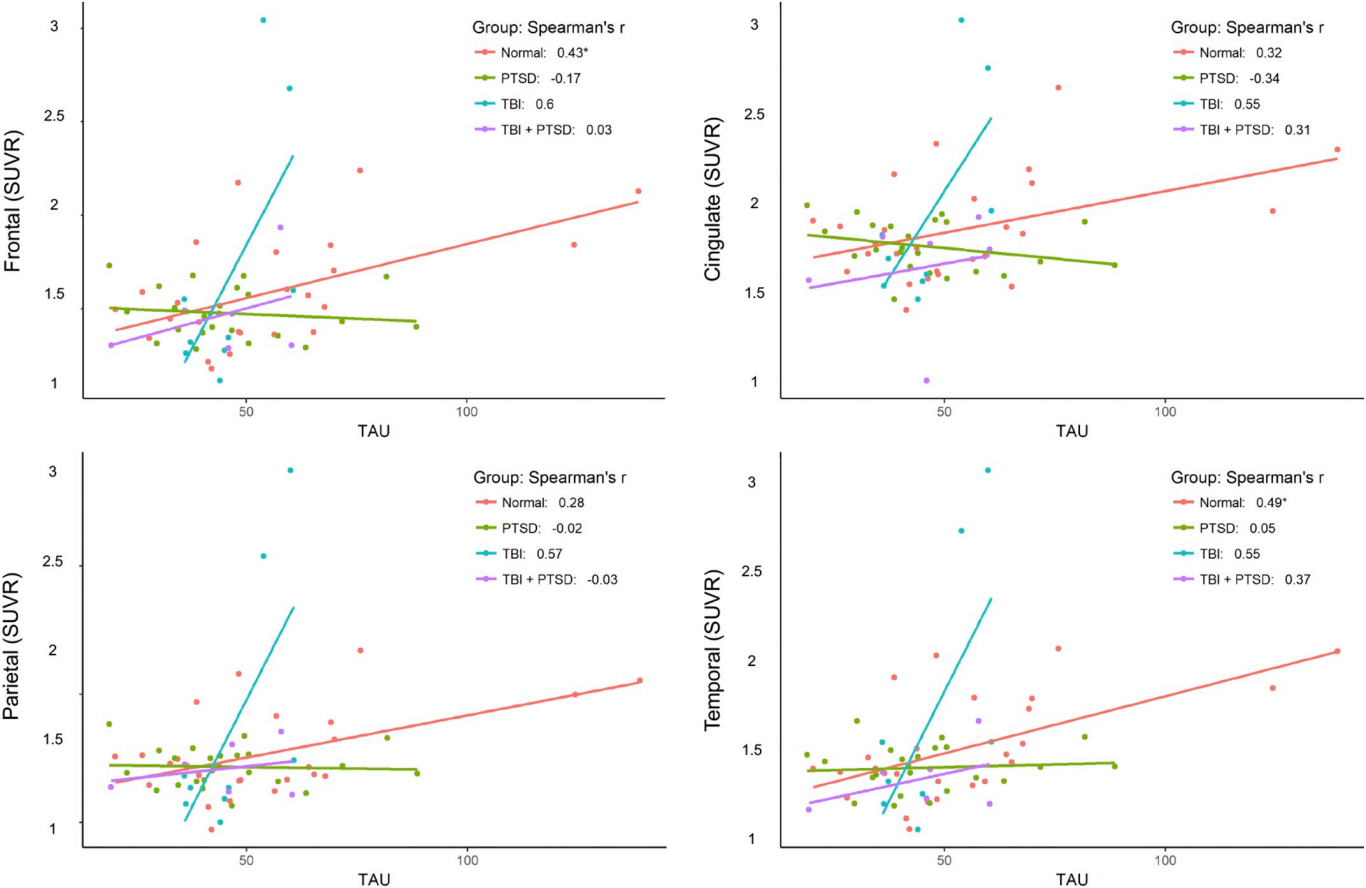

Fig. 7 shows the correlation between SUVR in different lobes of the brain and the tau concentration in cerebrospinal fluid. Significant positive correlations observed in all regions for TBI and only in frontal lobe for TBI_PTSD.

## 4 Discussion

The following statement should be added to the end of section 4.2.:

To further illustrate the findings of our voxel-based analysis, we defined clusters of interest in the right and left temporal lobe based on the difference map between PTSD and healthy controls, according to a statistical threshold of 0.01 < p < 0.0001. These clusters of interest showed small but statistically significant focal elevations of SUVR in the PTSD group compared to controls, while the whole temporal lobe region of interest showed no significant differences between groups. Thus, as might be expected given the focal increases in amyloid PET signal within the temporal lobe, averaging the signal from voxels in the entire temporal lobe dilutes this difference, resulting in no difference at the lobar level. Therefore, the finding by Weiner et al. of no group differences for entire cerebral cortex likely reflects this dilution of focal differences. We suppose that their global approach may be better suited for detecting widespread amyloid deposition in Alzheimer's disease than in trauma patients without dementia.

The authors would like to apologise for any inconvenience caused.

